# Thiopurines Induce Oxidative Stress in T-Lymphocytes: A Proteomic Approach

**DOI:** 10.1155/2015/434825

**Published:** 2015-03-22

**Authors:** Misbah Misdaq, Sonia Ziegler, Nicolas von Ahsen, Michael Oellerich, Abdul R. Asif

**Affiliations:** ^1^Institute of Clinical Chemistry/UMG Laboratories, University Medical Centre Goettingen, 37075 Goettingen, Germany; ^2^Medical Laboratories, 28357 Bremen, Germany

## Abstract

Thiopurines are extensively used immunosuppressants for the treatment of inflammatory bowel disease (IBD). The polymorphism of thiopurine S-methyltransferase (TPMT) influences thiopurine metabolism and therapy outcome. We used a TPMT knockdown (kd) model of human Jurkat T-lymphocytes cells to study the effects of treatment with 6-mercaptopurine (6-MP) and 6-thioguanine (6-TG) on proteome and phosphoproteome. We identified thirteen proteins with altered expression and nine proteins with altered phosphorylation signals. Three proteins (THIO, TXD17, and GSTM3) with putative functions in cellular oxidative stress responses were altered by 6-TG treatment and another protein PRDX3 was differentially phosphorylated in TPMT kd cells. Furthermore, reactive oxygen species (ROS) assay results were consistent with a significant induction of oxidative stress by both TPMT knockdown and thiopurine treatments. Immunoblot analyses showed treatment altered expression of key antioxidant enzymes (i.e., SOD2 and catalase) in both wt and kd groups, while SOD1 was downregulated by 6-TG treatment and TPMT knockdown. Collectively, increased oxidative stress might be a mechanism involved in thiopurine induced cytotoxicity and adverse effects (i.e., hepatotoxicity) and an antioxidant cotherapy might help to combat this. Results highlight the significance of oxidative stress in thiopurines' actions and could have important implications for the treatment of IBD patients.

## 1. Introduction

Thiopurines (e.g., azathioprine (AZA), 6-mercaptopurine (6-MP), and 6-thioguanine (6-TG)) are purine analogues that induce immunosuppression and reduce proliferation of cancerous cells [1]. Different mechanisms of action have been reported for thiopurines including blocking replication by incorporation into DNA and transcription by incorporation into RNA, blocking Rac-1-mediated signal transduction, and an antimetabolic effect through inhibition of GTP synthesis by 6-methyl thioinosine monophosphate (6-MeTIMP) [2].

Thiopurines are prodrugs which undergo extensive metabolism in order to exert their cytotoxic action [3]. The complex metabolism of these agents has been extensively investigated in an attempt to elucidate their mechanisms of action for efficacy and toxicity [3]. There are three competing thiopurine metabolic pathways, that is, conversion to 6-thioguanine nucleotides (6-TGN) by hypoxanthine guanine phosphoribosyl transferase (HPRT), inosine monophosphate dehydrogenase (IMPDH), and various kinases and reductases, methylation by the polymorphic enzyme, thiopurine S-methyltransferase (TPMT), and catabolism to thiouric acid by xanthine oxidase (XO) [3]. The 6-TGN are incorporated into DNA which after nonenzymatic methylation by S-adenosylmethionine are converted to 6-meTGN [4]. During replication instead of cytosine, 6-meTGN preferentially base pairs with thymine [4]. The 6-meTGN:T base pairs resemble replication errors and provoke processing by mismatch repair (MMR) and result in cell death [4].

TPMT catalyzes the S-methylation of aromatic compounds; it has no known endogenous substrate and thiopurine drugs are its only known substrates [5]. TPMT enzyme activity exhibits a trimodal distribution in erythrocytes [6]. TPMT gene polymorphisms lead to an almost 50-fold variation in enzyme activity between individuals [7]. Variations in response to thiopurine drug therapy are mainly caused by TPMT genetic polymorphism (reviewed in [8]). Adverse effects of 6-MP/AZA include bone marrow suppression which is of major concern, occurring in 2–5% of inflammatory bowel disease (IBD) patients treated with thiopurines [9]. The risk of thiopurine induced myelosuppression is increased in patients with TPMT deficiency. Homozygous deficiency occurs in 0.3% (with very low or absent levels) and heterozygosity occurs in 11% (associated with intermediate levels) of the general population [10]. Liver toxicity occurs in 3–10% of AZA exposed patients with hypersensitivity, an idiosyncratic cholestatic reaction, or endothelial cell damage and results in drug withdrawal [11]. A number of different factors have been reported to be linked to this thiopurine induced hepatotoxicity including higher concentrations of methylated metabolites and mitochondrial injury associated with glutathione depletion [12].

Thiopurines are known to induce oxidative stress, especially in mitochondria [13], resulting in mitochondrial dysfunction and activation of stress activated protein kinase pathways [14]. AZA induced oxidative stress causes tricarboxylic acid cycle dysfunction by depleting crucial mitochondrial enzymes [15]. 6-TGN is also known to incorporate into mitochondrial DNA (mtDNA) where it is rapidly oxidized and inhibits mtDNA replication. This leads to decreased mitochondrial protein concentrations and loss of mitochondrial function [16]. A recent study in cultured human lymphoblasts proposed ROS generation, causing oxidative DNA damage and mitochondrial dysfunction as the mechanism responsible for thioguanine induced cytotoxicity [17]. Gene expression studies have also reported thiopurine induced alterations in the expression of genes involved in protein and ATP-biosynthesis [18]. When mice were treated with 6-MP, significant alterations were observed in the expression of genes associated with abnormal lipid metabolism, inflammatory responses, oxidative stress, ATP depletion, and cell death [19].

In the study reported here we employed a proteomic approach to identify cellular targets of thiopurine therapy and differential TPMT activity. Stable TPMT knockdown (>80% reduction in protein level and 72% in enzyme activity level) was established in cultured human T-lymphocytes using sequence specific shRNAs [20]. These TPMT knockdown cells were then used for the first time as a model in a proteomic profiling study. We reasoned that the functional association of regulated proteins might be helpful in understanding the effects of thiopurine therapy on crucial cellular processes or identifying new cellular targets or potential approaches to the individualized therapy with thiopurine drugs.

## 2. Methods

### 2.1. Reagents

All cell culture reagents, RPMI, fetal calf serum (FCS), phosphate buffered saline (PBS), penicillin, and streptomycin, were purchased from PAA Laboratories, Colbe, Germany. Acetonitrile (ACN) was purchased from Promochem, Wesel, Germany. CHAPS was obtained from Applichem, Darmstadt, Germany. Urea, thiourea, dithiothreitol (DTT), trypsin, trifluoroacetic acid (TFA), sodium carbonate, ammonium bicarbonate, 6-MP, 6-TG, and DMSO were obtained from Sigma-Aldrich, Steinheim, Germany. Ampholytes, protein assay kits, and immobilized pH gradient strips (IPG strips) were obtained from Bio-Rad, Munich, Germany. Protease and phosphatase inhibitor cocktails were purchased from Roche, Mannheim, Germany. Glycerin, potassium ferricyanide, and sodium thiosulfate were purchased from Merck, Darmstadt, Germany. Formic acid was from BASF, Ludwigshafen, Germany. Bromophenol blue and Trizma base were from Carl Roth, Karlsruhe, Germany. Sodium dodecyl sulfate (SDS) was purchased from Serva, Heidelberg, Germany.

### 2.2. Cell Cultures

The thiopurines are immunosuppressive drugs that target T-lymphocytes, so we chose Jurkat cell line (T-lymphocytes) for our study. The wild-type Jurkat cell line (wt) was obtained from the German Collection of Microorganisms and Cell Cultures (DSMZ), Braunschweig, Germany. Cells were maintained in RPMI supplemented with 10% (v/v) heat-inactivated FCS, 100 U/mL penicillin, and 0.1 mg/mL streptomycin at 37°C in 95% humidity, 20% O_2_, and 5% CO_2_ in 75 cm^2^ culture flasks (Sarstedt, Nuembrecht, Germany). The Jurkat knockdown (kd) cells were maintained under hygromycin (Cayla, France) induced selective pressure (200 *μ*g/mL). To avoid any effect of selective agent on experimental findings, cells were seeded 24 hrs before thiopurine treatment and kept without hygromycin till the end of experiment. To ensure the same level of TPMT knockdown throughout study, cultures were frequently renewed with frozen cells of earlier passages. Further, the TPMT level of the running cultures was confirmed using real-time PCR or enzyme activity assays.

### 2.3. Sample Preparation for Proteome Analysis

Jurkat wt and kd cells were treated for 48 h with DMSO or IC_60_ doses of 6-MP (4.6 *μ*mol/L and 4.7 *μ*mol/L for wt and kd, resp.) or 6-TG (2.7 *μ*mol/L and 0.8 *μ*mol/L for wt and kd, resp.). The IC_60_ values were determined as described previously [20]; briefly cells were treated with DMSO (control) or 0.75 *μ*mol/L to 10 *μ*mol/L 6-MP and 0.25 *μ*mol/L to 8 *μ*mol/L 6-TG for 48 h. After incubation viability of the cells was estimated using microculture tetrazolium salts (MTS) reagent (Promega Corporation, Madison, WI) and IC_60_ values were calculated using GraphPad Prism Software version 5.01 (GraphPad Software, Inc.). Cells were harvested and washed three times with ice cold PBS. Washed cell pellets were obtained with centrifugation at 1800 rpm for 10 min at 4°C and then lysed in a buffer containing 7 mol/L urea, 2 mol/L thiourea, 4% w/v CHAPS, 2% ampholyte (pH 3–10), 1% DTT, 15% v/v protease, and 10% v/v phosphatase inhibitor cocktail. The protein content of lysates was estimated by Bradford assay using the Bio-Rad protein reagent according to the manufacturer's instructions. Sample aliquots were kept at −80°C until further use.

### 2.4. Two-Dimensional Electrophoresis (2-DE)

The 2-DE was performed as described previously [21] with some minor modifications. Briefly, protein samples (130 *μ*g) from Jurkat wt or kd cells were mixed with rehydration buffer (7 mol/L urea, 2 mol/L thiourea, 4% CHAPS, 0.2% ampholyte (pH 3–10), and 0.2% DTT) containing a trace amount of bromophenol blue to a total volume of 330 *μ*L. For rehydration (passive), samples were applied to nonlinear IPG strips (pH 3–10) for 1 h, then covered with mineral oil, and allowed to stand overnight at room temperature. Then isoelectric focusing (IEF) was performed in a Protean IEF cell (Bio-Rad) with the following configuration: 1 h at 100 volts, 1 h at 500 volts, and 2 h at 1000 volts and at 8000 volts with a total of 32000 volts-h. Before the second dimension electrophoretic separation, focused IPG strips were equilibrated for 30 min at room temperature in a buffer containing 50 mmol/L Tris-HCL (pH 8.8), 6 mol/L urea, 30% v/v glycerol, 2% SDS, and 10 g/L DTT followed by incubation in identical buffer in which DTT was replaced with 40 g/L iodoacetamide. The proteins in the equilibrated strips were then resolved on 12.5% SDS-PAGE in a Protean II chamber (Bio-Rad) at 100 V and 4°C.

### 2.5. Phosphospecific Staining of 2-DE Gels

Gel fixation was performed twice in a solution containing 50% methanol and 10% acetic acid for 45 minutes each followed by three 15 min washes in double distilled water. The gels were stained with Pro-Q Diamond phosphostain (Invitrogen, Paisley, UK) overnight in the dark, at room temperature. Destaining was done three times for 30 min in destaining solution containing 20% ACN and 50 mmol/L sodium acetate, followed by three washes in double distilled water each for 5 min. The gels were scanned using an imaging instrument (FLA-5100 Fuji photo film, Dusseldorf, Germany) at a wavelength of 532 nm. For excision of phosphorylated proteins from silver gels, the phospho- and silver gels (master gels in each case) were overlapped (warped) by using Delta 2D software (Decodon GmbH, Greifswald, Germany). The landmarks were then transferred from phospho- to silver gels. To ensure the exact localization, two independent individual assessments were also performed to confirm the software assisted spot position.

### 2.6. Protein Visualization: Densitometric and Statistical Analysis

Briefly, gels were fixed, followed by washing and sensitization. The gels were stained with freshly prepared silver nitrate solution (0.2% silver nitrate and 0.026% formaldehyde) for 20 min at room temperature followed by three 20 sec washes in double distilled water. Gels were immersed in developing solution containing 6% sodium carbonate, 0.0185% formaldehyde, and 6% sodium thiosulfate. When spots appeared, the reaction was stopped with stop solution (50% methanol and 12% acetic acid). After developing, the gels were scanned with a color image scanner Cano scan 8400 (Canon, Tokyo, Japan). Five independent 2-DE experiments were performed with each treatment and cell type and Delta 2D software version 3.6 (Decodon GmbH, Greifswald, Germany) was used for densitometric analysis [22].

After matching the spots across the gels, spot intensities were calculated and normalized by dividing the intensity of each spot by the sum of the intensity of all spots on the corresponding gel and the resultant intensity was considered to be the relative intensity of that particular spot. Microsoft Excel was used to calculate mean, standard deviation, ratio, fold change, and significance (using Student's* t*-test). Spots showing at least 1.5-fold expression changes (*P* ≤ 0.05) were considered statistically significant. GraphPad Prism Software version 5.01 (GraphPad Software, Inc.) was used to generate graphs.

### 2.7. Tryptic Digestion

Differentially expressed spots were excised from silver stained gels, followed by in-gel digestion according to the modified method earlier described by Shevchenko and colleagues [23]. Briefly, excised gel spots were washed with 30 mmol/L potassium ferricyanide and 100 mmol/L sodium thiosulfate for destaining, followed by washing with 50% ACN and 100 mmol/L ammonium bicarbonate and drying in a vacuum centrifuge (UNIVAPO, uniEquip, Martinsried, Germany). After drying, tryptic digestion was performed with trypsin digestion buffer (0.1 *μ*g/*μ*L trypsin, 1 mol/L calcium chloride, and 1 mol/L ammonium bicarbonate) on ice for 45 min and then incubated overnight in digestion buffer without trypsin at 37°C. The peptides were extracted with increasing concentrations of ACN and TFA and the extracted peptides were dried by vacuum centrifugation. Peptides were reconstituted in 0.1% FA for injecting into a nanoflow HPLC.

### 2.8. Peptide Sequence Analysis Using Nano-LC ESI Q-TOF MS/MS and Database Search

The peptide samples (1 *μ*L) were introduced onto two consecutive C18-reversed phase chromatography columns (C18 PepMap: 300 *μ*m × 5 mm; 5 *μ*m particle size, and C18 PepMap100 nanoanalytical column: 75 *μ*m × 15 cm; 3 *μ*m particle size; LC Packings, Germering, Germany) using a nanoflow CapLC autosampler (Waters, Eschborn, Germany). The peptides were eluted with an increasing gradient of ACN and analyzed on a Q-TOF Ultima Global mass spectrometer (Micromass, Manchester, UK) equipped with a nanoflow ESI Z-spray source in the positive ion mode, as previously described [24]. The data were analyzed with the MassLynx (version 4.0) software. The peak lists were searched using the online MASCOT search engine (http://www.matrixscience.com/) against the UniProt/SwissProt database release 15.15 (515203 entries, 181334896 elements). The data were searched against the database using the following parameters: trypsin as enzyme for digestion; up to a maximum of one missed cleavage site allowed; monoisotopic mass value and with unrestricted protein mass; peptide tolerance ±0.5 Da and MS/MS tolerance ±0.5 Da. Proteins were identified on the basis of two or more peptides whose ions score exceeded the threshold, and *P* ≤ 0.05 reflected the 95% confidence level for the matched peptides.

### 2.9. Functional Classification

Biological functions were assigned to the identified proteins using COGnitor (http://www.ncbi.nlm.nih.gov/COG/) [25].

### 2.10. Western Blotting

Briefly, proteins were separated on 12.5% SDS-PAGE and blotted onto PVDF membrane (Millipore, Schwalbach, Germany) using a semidry Trans-Blot system (SD Trans-Blot, Bio-Rad, Munich, Germany). The membrane was blocked with 5% skimmed milk powder prepared in TBS-T buffer (50 mmol/L Tris-HCl (pH 7.5), 200 mmol/L NaCl, and 0.05% Tween 20) for 1 h at room temperature. After washing three times with TBS-T buffer, the membrane was incubated with 1 : 1000 diluted; rabbit anti-stathmin (Cell Signaling Technology, Danvers, MA) or rabbit anti-SOD1 (Abnova, Taipei, Taiwan) or rabbit anti-SOD2 (Abcam, Cambridge, UK) or rabbit anti-CAT (Rockland, Gilbertsville, PA) and 1 : 10000 mouse beta-actin (Sigma-Aldrich, Steinheim, Germany) overnight at 4°C. After completion of incubation the membrane was washed and further incubated with appropriate HRP-conjugated secondary antibodies for 1 h at room temperature. Proteins were detected using an enhanced chemiluminescent (ECL) reagent (GE Healthcare, Munich, Germany). Blots were developed on Amersham Hyperfilm (GE Healthcare, Munich, Germany). Four independent experiments were performed for stathmin and signal intensities from each immunoblot were quantified using LabImage software version 2.71 (Kapelan, Leipzig, Germany). Three independent experiments were performed for SOD1, SOD2, and CAT.

### 2.11. ROS Assay

The level of reactive oxygen species was determined as described previously [26] with some modifications. After treatment of cells with IC_60_ doses of 6-MP and 6-TG for 48 h, media were removed by centrifugation. Cells were resuspended in PBS containing 10 *μ*mol/L DCFDA (Sigma-Aldrich, Steinheim, Germany) and incubated at 37°C for 1 h. The fluorescence intensity was determined with a Lambda Fluoro 320 fluorescence plate reader (MWG Biotech, Penzberg, Germany) with the excitation and emission wavelength settings at 485 and 530 nm, respectively. Finally, cell counting was done for each sample for normalization. The ROS assays were repeated in four independent cell preparations, each in triplicate.

## 3. Results and Discussion

**Figure 1:**
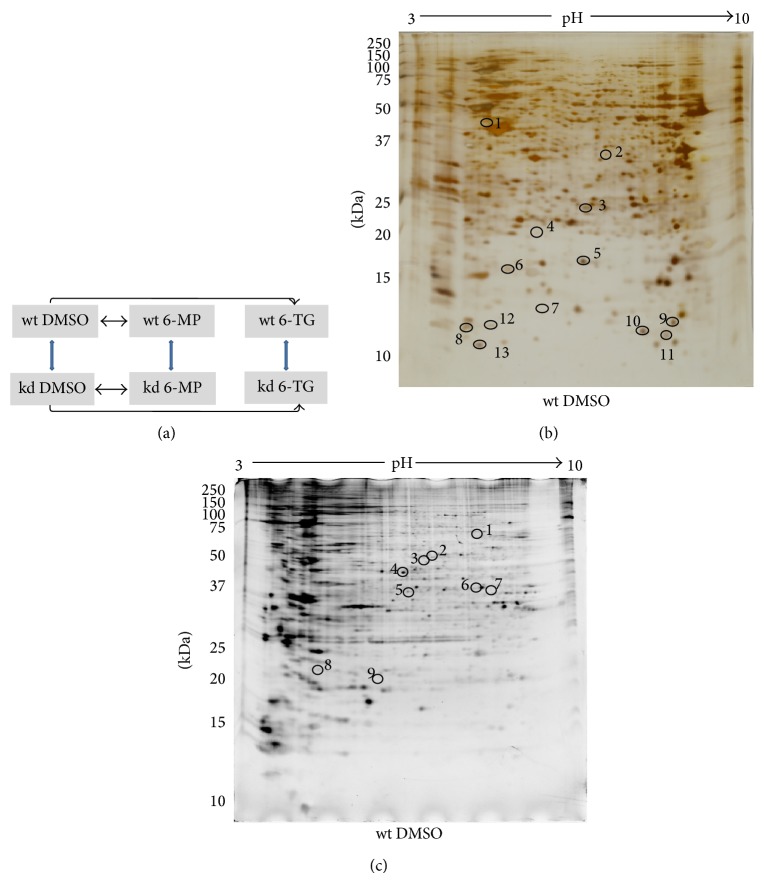
Schematic representation of experimental system. (a) Silver stained 2-DE gel (Jurkat wt DMSO), (b) phosphor stained 2-DE gel (Jurkat wt DMSO), and (c) Jurkat wt and kd cells were treated with IC_60_ doses of 6-MP, 6-TG, and vehicle (DMSO) for 48 h. Total protein lysates from DMSO, 6-MP, and 6-TG treated Jurkat wt and kd cells were separated by 2-DE, followed by staining with phosphor specific and silver stain. Consistently regulated (circled) spots were identified by Q-TOF MS/MS analysis. Densitometric analysis of Jurkat wt and kd groups (a); DMSO treated, silver stained, and phosphor stained representative gels of Jurkat wt ((b) and (c)) cell lysates.

The influence of thiopurine treatment on the proteome and phosphoproteome of human T-lymphocytes (Jurkat) with wild-type (wt) normal TPMT expression and knockdown (kd) cells with reduced TPMT expression was investigated. The cells were treated with vehicle DMSO or IC_60_ doses of 6-MP or 6-TG for 48 h (Figure 1(a)). After treatment, cells were harvested and lysed and total protein lysate was used for 2-DE. The gels were stained with phosphospecific staining followed by silver staining (see supplementary Figure  1 of the Supplementary Material available online at http://dx.doi.org/10.1155/2014/434825). The differentially regulated spots with ≥±1.5-fold changes (*P* ≤ 0.05 as determined by Student's* t*-test) were excised, digested, and identified by Q-TOF MS/MS analysis. Statistical analysis indicated that a total of thirteen proteins showed significant altered expression and nine proteins showed altered phosphorylation (Figures 1(b) and 1(c)). Predicted and actual pI of proteins, as well as molecular masses with their SwissProt accession numbers, and MS/MS spectral information is provided in Supplementary Tables 1 and 2. Significant changes were observed at the proteome and phosphoproteome levels, as a result of both TPMT knockdown and thiopurine treatment. Interestingly 6-MP and 6-TG showed different protein regulation patterns in both cell types. The identified proteins with significantly altered expression and phosphorylation are involved in diverse cellular functions with the majority of proteins involved in three functional categories, that is, oxidative stress response, cell cycle regulation, and regulation of cytoskeleton dynamics (Tables 1 and 2).

**Figure 2:**
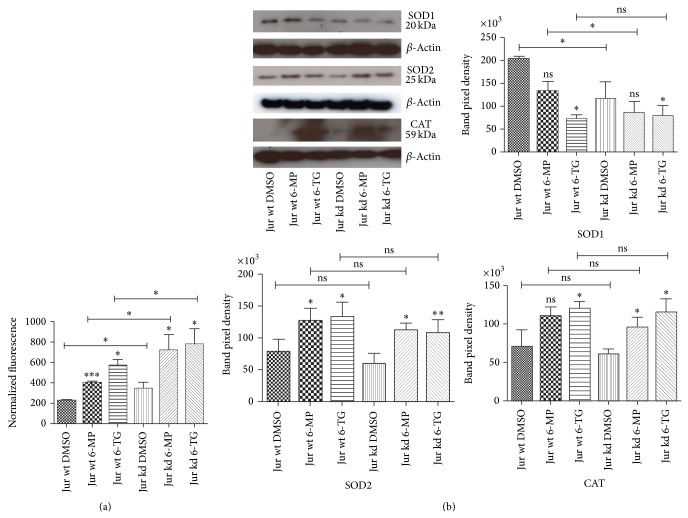
ROS assay after 6-MP or 6-TG treatment (a) and differential expression of ROS related SOD1, SOD2, and CAT proteins (b). (a) Cells were treated with vehicle DMSO or IC_60_ doses of 6-MP or 6-TG for 48 h. After treatment media were removed and the cells were resuspended in PBS containing 10 *μ*mol/L DCFDA. Fluorescence intensity was measured after 1 h incubation at 37°C. The error bars represent mean ± SD of four independent experiments (in triplicate format each). ^*^ *P* ≤ 0.05; ^***^ *P* ≤ 0.0005. For expressional regulation of ROS related proteins, total protein lysates from DMSO or 6-MP or 6-TG treated Jurkat wt and kd cells were separated by 1D gel electrophoresis and immunoblotted with antibody against SOD1, SOD2, and CAT (b). Beta-actin was used as a loading control.

Based on these findings, we further assessed cellular oxidative stress using ROS assays (Figure 2(a)). Intensity of fluorescence generated by incubating treated and control cells with the fluorescence dye DCFDA was measured and normalized to total cell number. The results showed significantly increased fluorescence intensity in wt and kd cells as a result of 6-MP and 6-TG treatment compared to their DMSO treated controls. All TPMT knockdown groups showed higher ROS levels compared to their wild-type counterparts. In order to see the effects of ROS accumulation on cellular antioxidant machinery, we then measured the alterations in the expression of three important antioxidant enzymes, that is, superoxide dismutase 1 (SOD1), superoxide dismutase 2 (SOD2), and catalase (CAT) by Western blot analysis (Figure 2(b)).

### 3.1. Proteins Regulated by TPMT Knockdown

Comparison of Jurkat kd cells to their wt counterparts when grown in DMSO, 6-MP, or 6-TG showed altered expression of six proteins in kd cells (Table 1; Figure 1(b) shows a representative silver stained gel of wt DMSO with these spots marked). Of these proteins, four were significantly upregulated (spots 2–5) and two were downregulated (spots 1 and 13). Heterogeneous ribonucleoproteins H3 (HNRH3), delta(3, 5)-delta(2, 4)-dienoyl-CoA isomerase (ECH1), deoxyuridine 5′-triphosphate nucleotidohydrolase, mitochondrial (DUT), and cofilin 1 (COF1) were upregulated. Ataxin 10 (ATX10, spot 1) was downregulated in kd DMSO cells compared to wt DMSO. Myotrophin (MTPN, spot 13) was downregulated in kd 6-TG cells compared to wt 6-TG. COF1 is a key player in actin dynamics and has emerged as an agent of cellular homeostasis [27]; its upregulation in kd (DMSO, 6-MP, and 6-TG) cells indicates the importance of TPMT in cellular homeostatic processes. MTPN is a multifunction, ankyrin repeat protein which is ubiquitously expressed in all mammalian tissues [28]. COF1 and MTPN are involved in actin dynamics and polymerization, respectively. Their altered expression in kd cells indicates the possible involvement of TPMT or its substrates in cytoskeleton regulation. COF1 has a proapoptotic role; the mitochondrial translocation of COF1 induces cytochrome c release and apoptosis [29]. ROS also influence COF1 activity by promoting its dephosphorylation [30]. Hence, the known role of COF1 in cytoskeletal changes induced by oxidative stress [30] might also be involved in the mechanism of action of thiopurines (Supplementary Figures 2(a) and 5(a)). Densitometric analysis of phosphostained gels identified six proteins with altered phosphorylation signals (Table 2; Figure 1(c) shows a representative phosphostained gel of wt, DMSO treated cells with these spots marked). Of these six proteins, four exhibited increased phosphorylation as a result of knockdown (i.e., 6-phosphofructokinase type C (K6PP, spot 1), T-complex protein 1 subunit zeta (TCPZ, spot 2), glutathione S-transferase Mu 3 (GSTM3, spot 8), and thioredoxin-dependent peroxide reductase (PRDX3, spot 9)). TCPZ is a subunit of TCP-1 (CCT) which contains chaperonin and is involved in the production of native actin, tubulin, and numerous other proteins required for cell cycle progression [31]. We observed an increased phosphorylation of TCPZ in 6-MP treated kd cells compared to 6-MP treated wt cells (Supplementary Figures 2(b) and 6(a)). Altered phosphorylation of this TCPZ subunit possibly indicates a change in the affinity of CCT towards substrate proteins as a result of 6-MP treatment of TPMT deficient cells.

GSTM3 is a member of the glutathione S-transferase (GST) family. It protects cells from oxidative stress by neutralizing reactive compounds [32]. Posttranslational modifications such as serine, threonine, or tyrosine phosphorylation can modulate GST stability and function [33]. GST is known for its role in conjugation of AZA with glutathione, a step required for its conversion to 6-MP. A correlation between GST polymorphism and AZA adverse effects has also been reported [34]. In this study, 6-TG treatment caused increased GSTM3 phosphorylation in both wt and kd cells which might attenuate its function and in turn influence oxidative stress in cells. PRDX3 (thioredoxin peroxidase-3) is localized in mitochondria and uses mitochondrial thioredoxin-2 (Trx2) as an electron donor to scavenge H_2_O_2_ [35]. PRDX3 phosphorylation is associated with decreased peroxidase activity, increased oxidative stress, and consequently dysregulation of mitochondrial function [36]. We also observed upregulation of PRDX3 in kd cells (Supplementary Figures 3 and 6(b)). Increased GSTM3 and PRDX3 phosphorylation along with SOD1 downregulation in TPMT treated kd cells correlate with the oxidative stress suggested by the ROS assay results. SOD1 catalyzes the conversion of the superoxide radical to hydrogen peroxide which is further cleared by CAT [37]. Downregulation of SOD1 is known to be associated with oxidative stress [38]. Similarly, upregulated expression of SOD2 and CAT after 6-MP and 6-TG treatment is also indicative of cellular responses to high oxidative stress and their upregulation could have a protective effect [39] because SOD2 helps to maintain ROS balance and produce a more ideal metabolic environment for the cell [40]. In kd cells oxidative stress was significantly higher compared to wt cells, with and without treatment. Since the natural substrate of TPMT is still not known, it can be hypothesized that TPMT knockdown might affect metabolism of some endogenous compound(s) which could lead to alteration in the expression or posttranslational modifications of antioxidant proteins and consequently increased oxidative stress.

Both the Rab GDP dissociation inhibitor beta (GDIB, spot 4) and the mRNA export factor (RAE1L, spot 7) showed decreased phosphorylation in kd cells. Rab GDP dissociation inhibitors bind prenylated Rab-GTPases, deliver Rab proteins to specific compartments, and retrieve Rabs from fusion targets after completion of the catalytic cycle [41]. TPMT knockdown decreased phosphorylation of GDIB. Previous studies showed that phosphorylation of GDI might be associated with more effective retrieval of Rab proteins from membranes (reviewed in [41]). Decreased GDIB phosphorylation might indicate an alteration in GDIB activity which could consequently modulate cell cycle progression. Eukaryotic cells regulate fundamental cellular processes by rapid destruction of key regulatory proteins, such as cell cycle regulators and transcription factors, catalyzed by PRS10 [42]. Studies on yeast showed decreased PRS10 ATPase activity after dephosphorylation and vice versa [43]. We observed decreased PRS10 phosphorylation in this study.

RAE1L is a shuttling transport factor that permits efficient export of mRNA through the nuclear pore complex (NPC) [44]. Its phosphorylation was increased as a result of 6-TG treatment in kd cells, but compared to wt 6-TG its signal was weaker. NPC dynamics play a critical role during cell cycle transitions (reviewed in [45]). Phosphorylation of RAE1L and some other proteins of the nuclear complex by mitotic kinases results in altered NPC dynamics (reviewed in [44]).

### 3.2. Proteins Regulated by 6-MP (4.6 *μ*mol/L) and 6-TG (2.8 *μ*mol/L) Treatment in Jurkat wt Cells

**Figure 3:**
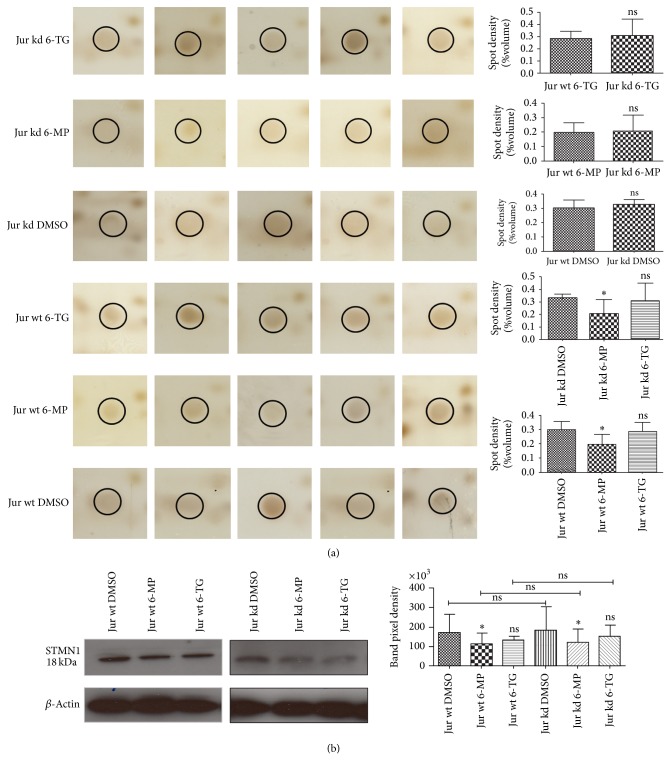
Differential expression of STMN1 as shown by silver stained gels (a) and Western blot analysis (b). (a) STMN1 spot in all six groups: significant downregulation in wt and kd 6-MP treated groups. Graphical representation of spot density (% volume) with mean ± SD of five independent experiments (^*^ *P* ≤ 0.05). (b) Total protein lysates from DMSO or 6-MP or 6-TG treated Jurkat wt and kd cells were separated by 1D gel electrophoresis and immunoblotted with antibody against STMN1. Densitometric analyses were performed using LabImage 2.71 software. Beta-actin was used as a loading control. The error bars represent mean ± SD of four independent experiments (^*^ *P* ≤ 0.05).

Jurkat wt cells were treated with IC_60_ doses of 6-MP (4.6 *μ*mol/L) and 6-TG (2.8 *μ*mol/L). Comparison of 6-MP treated Jurkat wt cells with their DMSO controls showed significant expression changes in three proteins (Table 1). Spot 6, corresponding to stathmin 1 (STMN1), was downregulated (Figure 3(a) and Supplementary Figure 5(c)). Regulation of STMN1 was further confirmed by Western blot analysis (Figure 3(b)). Spot 7, identified as costars family protein ABRACL (ABRAL), was also significantly downregulated in 6-MP treated wt cells. Spot 11, hemoglobin subunit alpha (HBA), was upregulated as a result of 6-MP treatment in wt cells (Supplementary Figures 4(a) and 5(b)). Thiopurines are known to induce cell death by inserting mismatches in DNA and activating cell cycle check points (ATM/Chk2 and ATR/Chk1) which result in cell cycle arrest at G2/M phase and cell death [4]. STMN1 is a phosphoprotein that acts as a microtubule regulator during mitosis [46]; its expression was downregulated by 6-MP treatment. Inhibition of STMN1 expression is associated with severe mitotic spindle abnormalities and difficulty in exiting from mitosis that leads to the accumulation of cells in the G2/M phases. These results are consistent with previously reported G2/M phase cell arrest caused by thiopurines (reviewed in [4]). The mechanism responsible for this cell cycle arrest is DNA strand breaks (reviewed in [4]). However, downregulation of STMN1 might also be a mechanism responsible for the G2/M phase arrest caused by thiopurines. The ABRAL, a member of the costars family, was downregulated by exposure to 6-MP. There is little functional information available for ABRAL (or HSPC280), but some studies indicate that it has a possible role in cell cycle regulation and DNA damage response [47, 48]. This finding correlates well with the thiopurine induced cell cycle disturbances.

6-TG treatment influenced expression of seven proteins in wt cells. There was upregulation of COF1, thioredoxin (THIO), profilin 1 (PROF1), macrophage migration inhibitory factor (MIF), hemoglobin subunit alpha (HBA), thioredoxin domain containing protein 17 (TXD17), and MTPN. These proteins corresponded to spots 5 and 8–13, respectively, and are marked in a representative gel (Figure 1(b)). THIO was upregulated in Jurkat wt cells by 6-TG treatment. It catalyzes reversible reduction of protein disulfide bonds (reviewed in [49]). It is primarily localized in the cytosol, but with oxidative stress it translocates to the nucleus (reviewed in [49]). THIO upregulation may be a response to 6-TG induced oxidative stress. PROF1 overexpression induces cell cycle arrest in breast cancer cells and sensitizes cells to apoptosis in response to a cytotoxic stimulus [50]. PROF1 upregulation observed here might also sensitize cells to 6-TG induced apoptosis.

Alteration of the phosphoproteome was also observed after 6-MP and 6-TG treatment. Five proteins showed significantly changed phosphorylation signals: two after 6-MP exposure and three after 6-TG (Table 2, Figure 1(c)). Coronin-1A (COR1A) or spot 3 showed significantly downregulated phosphorylation in the wt 6-MP group. Decreased phosphorylation was observed in the 26S protease regulatory subunit 10B (PRS10, spot 6). In the 6-TG group, actin related protein-2 (ARP2, spot 5) showed downregulation of phosphorylation, while RAE1L (spot 7) and GSTM3 (spot 8) showed upregulation of phosphorylation. The coronins influence actin dynamics by inhibiting Arp2/3 complex nucleation [51]. Studies of COR1B showed regulation of its interaction with Arp2/3 through phosphorylation by protein kinase C (PKC) [52]. ARP2 is related to actin and forms part of the Arp2/3 complex that controls actin dynamics [53]. Phosphorylation of the Arp2/3 complex is necessary for actin filament nucleation. We observed thiopurine induced downregulation of ARP2 phosphorylation. This downregulation might have caused reductions in Arp2/3 complex activity and thereby affected actin dynamics. COR1A and ARP2 influence Arp2/3 nucleation and activity, respectively. It can be hypothesized that thiopurine treatment might affect actin dynamics by manipulating Arp2/3 complex nucleation and activity. Collectively, the alterations observed in the regulation of actin dynamics might be involved in the cytotoxicity or induction of apoptosis by thiopurines (Supplementary Figures 4(b) and 6(c)).

All the protein targets described in our study are already well characterized, but we are reporting them for the first time as a result of thiopurine exposure in both wt and kd cells. Thiopurines induce cell cycle arrest by DNA damage and oxidative stress, as well as by altering these protein targets. Alternately there may be as yet unclear crosstalk between these pathways that needs to be further investigated.

### 3.3. Proteins Regulated by 6-MP (4.7 *μ*mol/L) and 6-TG (0.8 *μ*mol/L) Treatment in Jurkat kd Cells

In total three proteins were differentially regulated in Jurkat kd cells: one after 6-MP treatment and two after 6-TG treatment (Table 1). STMN1 was downregulated after 6-MP treatment. ECH1 was one of two proteins that were significantly upregulated in kd cells exposed to 6-TG. The second upregulated protein in kd cells was MIF. Quantitative analysis showed four proteins with altered phosphorylation: one in 6-MP and three in 6-TG exposed cells (Table 2). After 6-MP exposure TCPZ phosphorylation was increased compared to the DMSO exposed control kd cells. In kd cells exposed to 6-TG K6PP showed decreased phosphorylation, while RAE1L and GSTM3 showed increased phosphorylation. Figure 1(c) shows a representative phosphostained gel of the wt DMSO group with these spots marked.

The proteins identified include some important targets of cellular oxidative stress response, that is, THIO, TXD17, GSTM3, and PRDX3. Similarly, important regulators of actin cytoskeleton, that is, COF1 and PROF1, which are known to be sensitive to oxidative stress (i.e., COF1 activity and PROF1 expression are regulated by oxidative stress [27, 54]), were also identified in both wt and kd cells.

*In vivo* studies show that thiopurines induce toxicity through induction of apoptosis and by a mechanism that involves oxidative stress, mitochondrial injury, and ATP depletion that can lead to irreversible deenergization and cell death by necrosis [13, 55]. Collectively, these and our results suggest that increased oxidative stress is a possible cause of thiopurine induced adverse drug reactions (ADRs) including hepatotoxicity in IBD and acute lymphoblastic leukemia patients. Allopurinol (Allo) is an antioxidant that acts by inhibiting xanthine oxidase (XO) [12]. Combination therapy with allopurinol and thiopurines for IBD patients can lead to major clinical improvements, but at the same time it may increase the chances of myelotoxicity and opportunistic infections [12, 56]. Cotherapy of thiopurines and allopurinol has not been reported to be used for ALL patients although the incidence of thiopurine induced hepatotoxicity is also significant in ALL patients [57]. One logical strategy to prevent thiopurine induced liver toxicity is to utilize antioxidant cotherapy, with drugs that do not affect thiopurine metabolism and that could be used in all TPMT activity groups without dosage readjustments. There are anecdotal reports of the successful use of herbal antioxidant therapies in combination with thiopurines to avoid liver injury including a case report of a 34-year-old woman suffering from promyelocytic leukemia who prior to combined therapy was unable to tolerate maintenance therapy (methotrexate and 6-mercaptopurine) due to liver toxicity [58]. This patient reportedly was able to complete therapy and achieved normal liver function, when treated with silymarin (an herbal antioxidant product) along with maintenance therapy.

## 4. Conclusion

**Figure 4:**
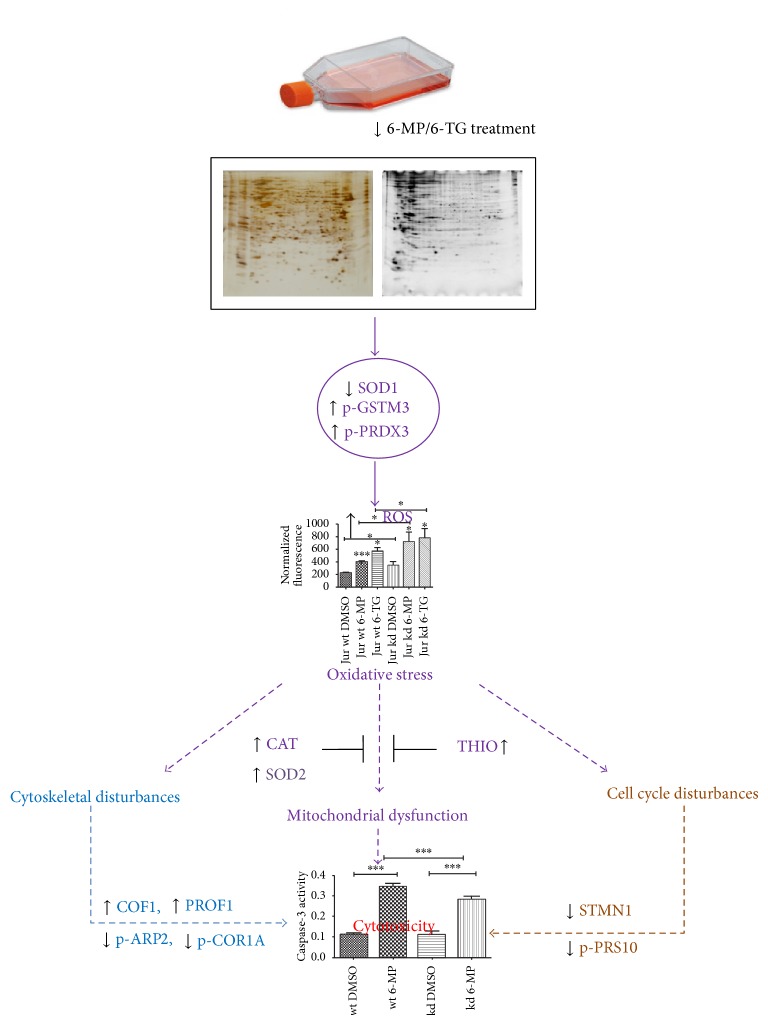
Thiopurine induced oxidative stress and proteome regulation 6-MP and 6-TG treatment induced phosphorylation changes in GSTM3 and PRDX3 (redox regulators of cell) which can consequently reduce their ROS neutralization activity [32, 36]. Similarly, TPMT knockdown and thiopurine treatment downregulated expression of SOD1 which together with altered activity of GSTM3 and PRDX3 might result in enhanced ROS accumulation. ROS assays showed an increase in ROS level after 6-MP and 6-TG treatment. Increased ROS levels may cause mitochondrial dysfunction [59]. Persistent and increasing mitochondrial dysfunction can induce cellular cytotoxicity and apoptosis [59]. On the other hand, to cope with increasing oxidative stress, cells could activate their cellular antioxidant mechanisms [59] as suggested by the increased expression of the antioxidant proteins THIO [60], SOD2 [61], and CAT [39]. ROS accumulation also affects cytoskeleton [62], suggested by the altered cytoskeleton regulator proteins (expression of COF1 and PROF1 and phosphorylation of p-ARP2, p-COR1A) [30, 50, 52, 53]. Oxidative stress influences the cell cycle [63], and the observed reduced expression of STMN1 (a regulator of microtubule dynamics during meiosis) and decreased phosphorylation of PRS10 (involved in ATP-dependent degradation of ubiquitinated proteins) may be indicative of this phenomenon [43, 46]. We hypothesize that 6-MP and 6-TG treatment affect the activity of antioxidant proteins which results in increased oxidative stress and consequently mitochondrial dysfunction, as well as cytoskeleton and cell cycle disturbances which collectively contribute to thiopurine induced cytotoxicity.

This is the first proteomics based study to investigate the effects of both thiopurine and differential TPMT activity on a cellular proteome. In both wild-type and knockdown cells, 6-MP and 6-TG treatment resulted in significant protein regulation. The regulated proteins identified were directly or indirectly involved in cellular responses to oxidative stress. Similarly, significant influence on cell cycle regulation and cytoskeleton reorganization mechanisms was identified. Taken together, these results suggest that thiopurine treatment can induce cytotoxicity by both DNA damage (a known mechanism of thiopurine action) and increased oxidative stress. Cell responses to these stresses include regulation of the expression and activity of important targets in the cell cycle and cytoskeleton systems. Both 6-MP and 6-TG affected the activity of antioxidant proteins which resulted in signs of increased oxidative stress and mitochondrial dysfunction, as well as cytoskeleton and cell cycle disturbances. These effects might contribute to thiopurine induced cytotoxicity in patients including myelotoxicity and hepatotoxicity (Figure 4). Protein targets with possible role in thiopurine induced oxidative stress and cytotoxicity were identified for the first time using proteomic and phosphoproteomic methods in both wild-type and knockdown human cell lymphocyte cell lines. Although the exact mechanism of these alterations is not known, further investigation may help to understand the complex mechanism of action of thiopurines or their prevention with antioxidant cotreatment in thiopurine treated patients.

## Supplementary Material

Supplementary Tables 1 and 2 contain MS/MS analysis data of differentially regulated proteins and phospho proteins. Supplementary material contains as Figure 1, silver and phospho stained 2-DE gels of all six experimental groups. Figures 2-4 contain spot diagrams of some differentially regulated proteins and phospho proteins along with spot density (% volume) graphs. Figures 5 and 6 contain fold change graphs of proteins represented in supplementary figures 2-4.

## Figures and Tables

**Table 1:** Differentially regulated proteins identified by mass spectrometry.

Spot number	Protein name^a^	Function (COGnitor NCBI and SwissProt)	Expression change (in fold)						
			Jurkat wt		Jurkat kd		kd DMSO/wt DMSO	kd 6-MP/wt 6-MP	kd 6-TG/wt 6-TG
			6-MP	6-TG	6-MP	6-TG			
	Oxidative stress								
8	Thioredoxin (THIO)	Cell redox homeostasis, protein thiol-disulfide exchange	NS	1.67^*^↑	NS	NS	NS	NS	NS
12	Thioredoxin domain containing protein 17 (TXD17)	Disulfide reductase, modulates TNF-alpha signaling and NF-kappa-B activation	NS	2.87^*^↑	NS	NS	NS	NS	NS

	Cell cycle regulation								
6	Stathmin 1 (STMN1)	Cell differentiation, microtubule depolymerization, intracellular signal transduction	1.53^*^↓	NS	1.53^*^↓	NS	NS	NS	NS
7	Costars family protein ABRACL (ABRAL)	Member of costars family	1.96^*^↓	NS	NS	NS	NS	NS	NS

	Cytoskeleton regulation								
5	Cofilin 1 (COF1)	Rho protein signal transduction, actin cytoskeleton organization, antiapoptosis	NS	2.53^*^↑	NS	NS	2.78^**^↑	1.5^*^↑	2.45^*^↑
9	Profilin 1 (PROF1)	Actin cytoskeleton organization, regulation of transcription from mRNA polymerase II promoter	NS	5.25^*^↑	NS	NS	NS	NS	NS
13	Myotrophin (MTPN)	Positive regulation of cardiac muscle hypertrophy, NF-kappa B transcription factor activity	NS	2.02^*^↑	NS	NS	NS	NS	1.75^*^↓

	Enzymes								
3	Delta (3,5)-delta(2,4)-dienoyl-CoA isomerase, mitochondrial (ECH1)	Involved in fatty acid metabolism and lipid metabolism	NS	NS	NS	2.22^***^↑	NS	NS	1.54^*^↑
4	Deoxyuridine 5′-triphosphate nucleotidohydrolase, mitochondrial (DUT)	Nucleotide metabolism, dUTP diphosphatase activity	NS	NS	NS	NS	2.02^**^↑	NS	1.78^*^↑

	Others								
1	Ataxin 10 (ATX10)	Role in the maintenance of a critical intracellular glycosylation level and homeostasis	NS	NS	NS	NS	2.63^*^↓	NS	NS
2	Heterogenous ribonucleoprotein H3 (HNRH3)	Nuclear mRNA splicing via spliceosome	NS	NS	NS	NS	1.74^*^↑	NS	NS
10	Macrophage migration inhibitory factor (MIF)	Cell proliferation, inflammatory response	NS	3.32^*^↑	NS	1.81^*^↑	NS	NS	NS
11	Hemoglobin subunit alpha (HBA)	Oxygen transport, hydrogen peroxide catabolic process	4.09^*^↑	5.55^*^↑	NS	NS	NS	NS	NS

^a^Proteins identified by Q-TOF MS/MS analysis and database search against SwissProt; ↑: upregulated; ↓: downregulated; NS: nonsignificant change.

^*^ *P* ≤ 0.05, ^**^ *P* ≤ 0.005, and ^***^ *P* ≤ 0.0005.

**Table 2:** Differentially regulated phosphoproteins identified by mass spectrometry.

Spot number	Protein name^a^	Function (COGnitor NCBI and SwissProt)	Phosphorylation change (in fold)						
			Jurkat wt		Jurkat kd		kd DMSO/wt DMSO	kd 6-MP/wt 6-MP	kd 6-TG/wt 6-TG
			6-MP	6-TG	6-MP	6-TG			
	Oxidative stress								
8	Glutathione S-transferase Mu 3 (GSTM3)	Glutathione transferase activity	NS	3.81^**^↑	NS	1.84^*^↑	NS	NS	2.26^*^↑
9	Thioredoxin-dependent peroxide reductase (PRDX3)	Involved in redox regulation of the cell	NS	NS	NS	NS	2.01^*^↑	2.14^***^↑	2.85^***^↑

	Cell cycle regulation								
2	T-complex protein 1 subunit zeta (TCPZ)	De novo posttranslational protein folding	NS	NS	1.86^**^↑	NS	NS	1.63^*^↑	NS
4	Rab GDP dissociation inhibitor beta (GDIB)	Regulates the GDP/GTP exchange reaction of most Rab proteins	NS	NS	NS	NS	1.53^*^↓	1.59^**^↓	NS
6	26S protease regulatory subunit 10B (PRS10)	ATP-dependent degradation of ubiquitinated proteins	1.56^**^↓	NS	NS	NS	NS	NS	NS
7	mRNA export factor (RAE1L)	Involved in nucleocytoplasmic transportof mRNA	NS	1.69^**^↑	NS	1.8^*^↑	NS	NS	1.85^*^↓

	Cytoskeleton regulation								
3	Coronin-1A (COR1A)	T-cell homeostasis, involved in cell locomotion	1.54^*^↓	NS	NS	NS	NS	NS	NS
5	Actin-related protein 2 (ARP2)	ATP-binding component of the Arp2/3 complex	NS	1.52^*^↓	NS	NS	NS	NS	NS

	Enzymes								
1	6-Phosphofructokinase type C (K6PP)	Involved in glycolysis, protein homotetramerization	NS	NS	NS	1.5^*^↓	1.65^*^↑	NS	NS

^a^Proteins identified by Q-TOF MS/MS analysis and database search against SwissProt; ↑: upregulated; ↓: downregulated; NS: nonsignificant change. ^*^ *P* ≤ 0.05, ^**^ *P* ≤ 0.005, and ^***^ *P* ≤ 0.0005.
