# Supplementary material for: Thiopurines Induce Oxidative Stress in T-Lymphocytes: A Proteomic Approach
Source: Mediators Inflamm. 2015 Mar 22;2015:434825. doi: 10.1155/2015/434825 (PMC4385670; doi:10.1155/2015/434825)
Supplement: Supplementary file 1 — Supplementary Tables 1 and 2 contain MS/MS analysis data of differentially regulated proteins and phospho proteins. Supplementary material contains as Figure 1, silver and phospho stained 2-DE gels of all six experimental groups. Figures 2-4 contain spot diagrams of some differentially regulated proteins and phospho proteins along with spot density (% volume) graphs. Figures 5 and 6 contain fold change graphs of proteins represented in supplementary figures 2-4. [file 434825.f1.pdf]

Suppl. Figure 1(a)

Jur wt DMSO

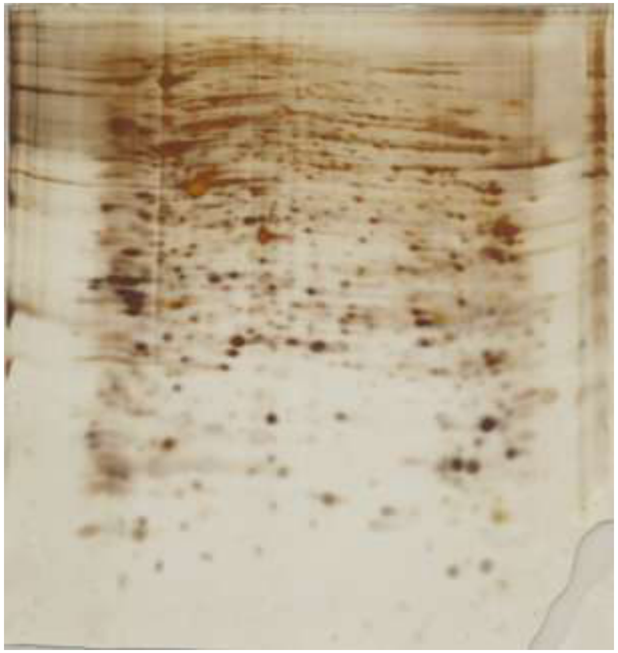

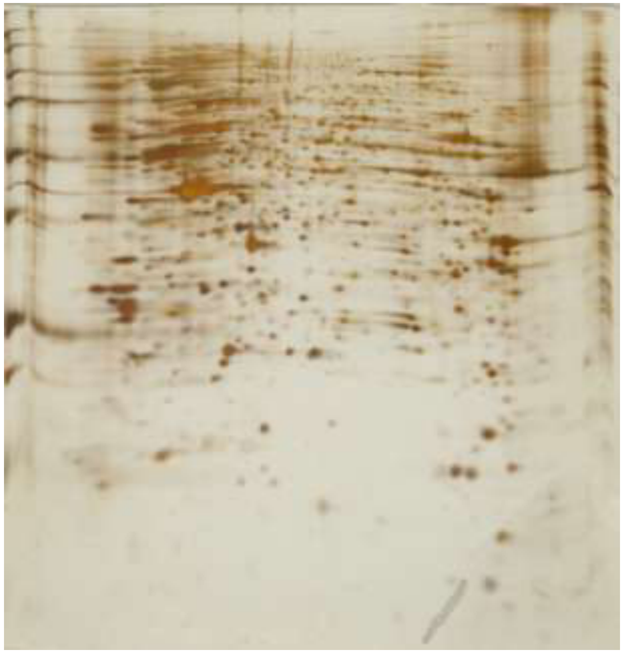

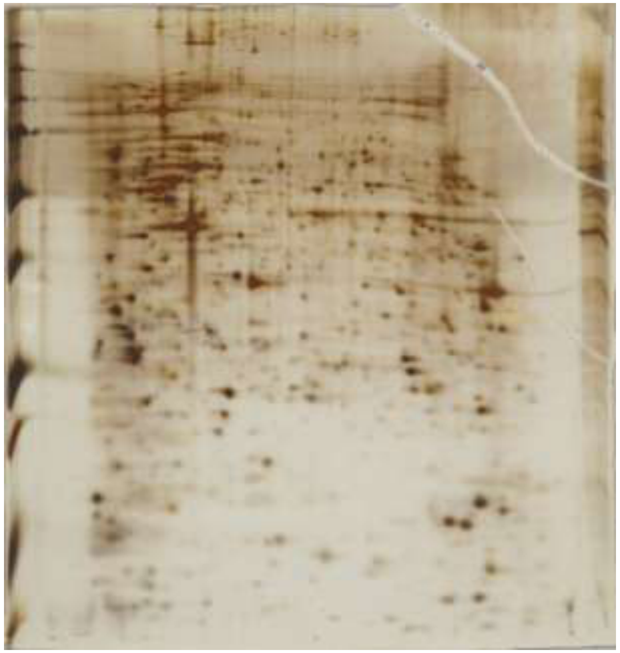

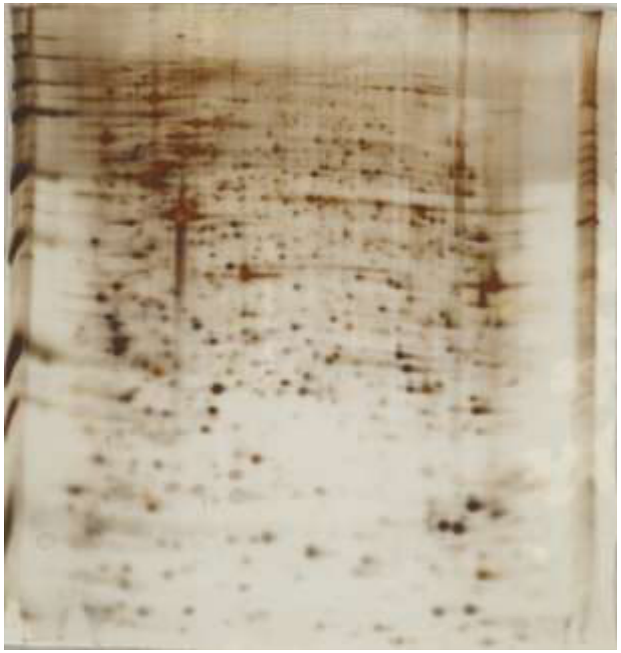

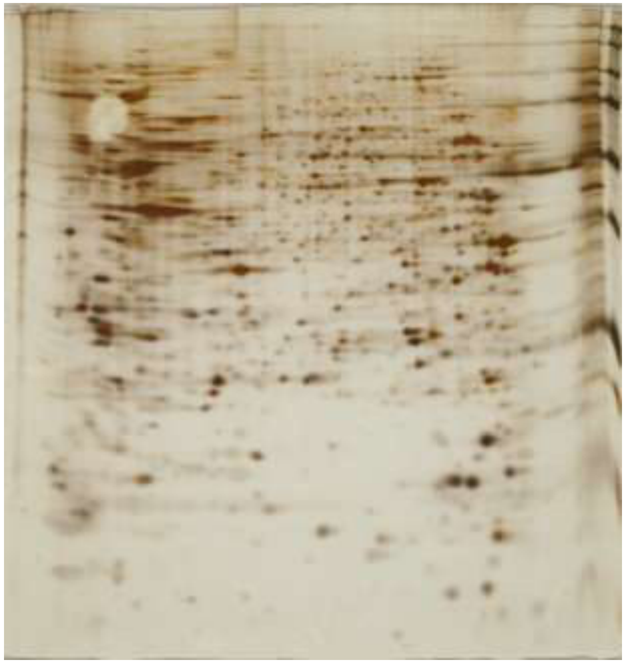

Jur wt 6-MP

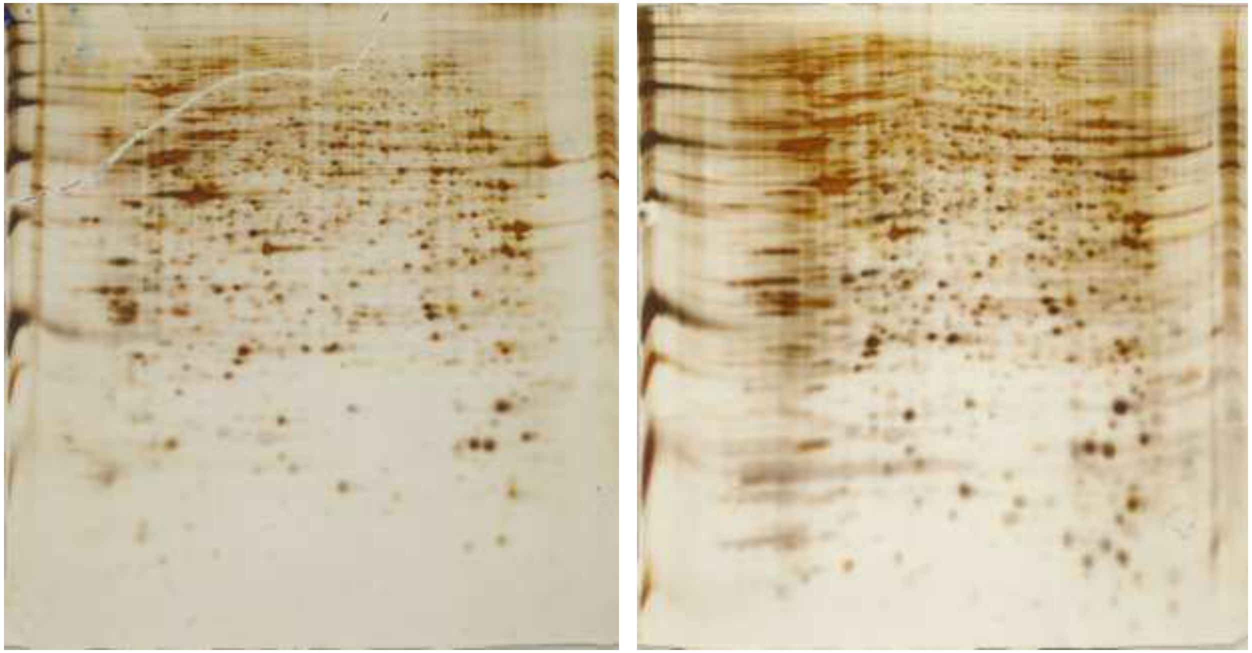

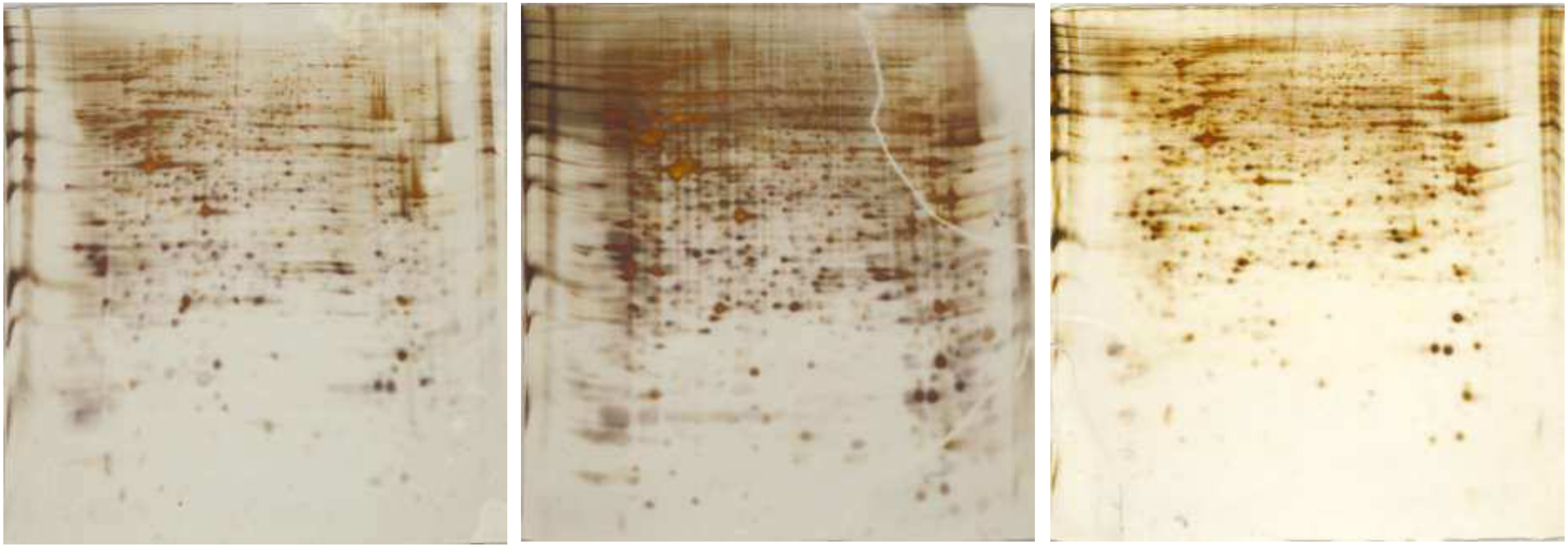

Jur wt 6-TG

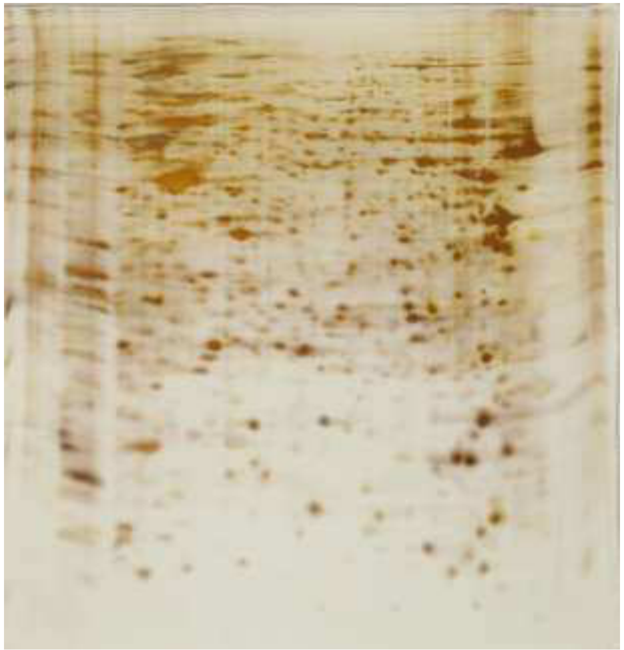
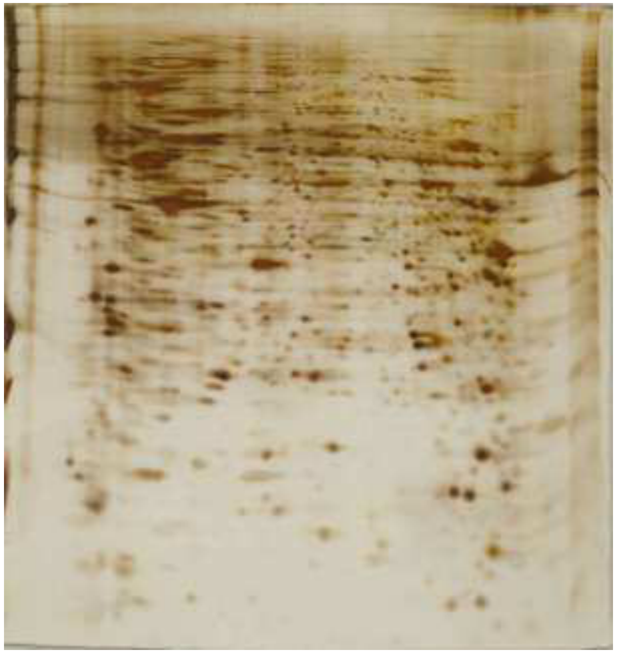
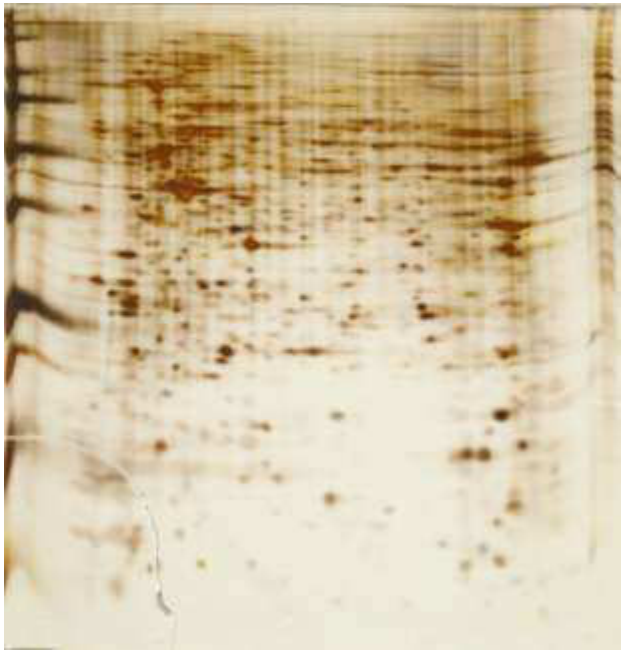
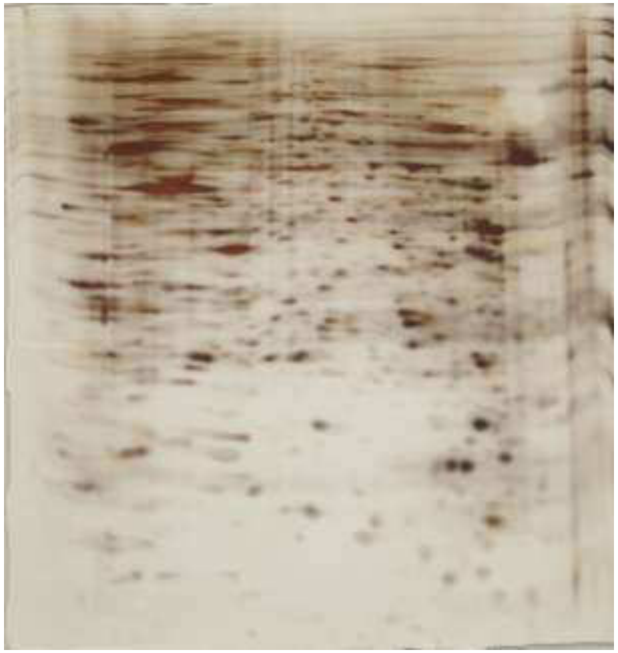
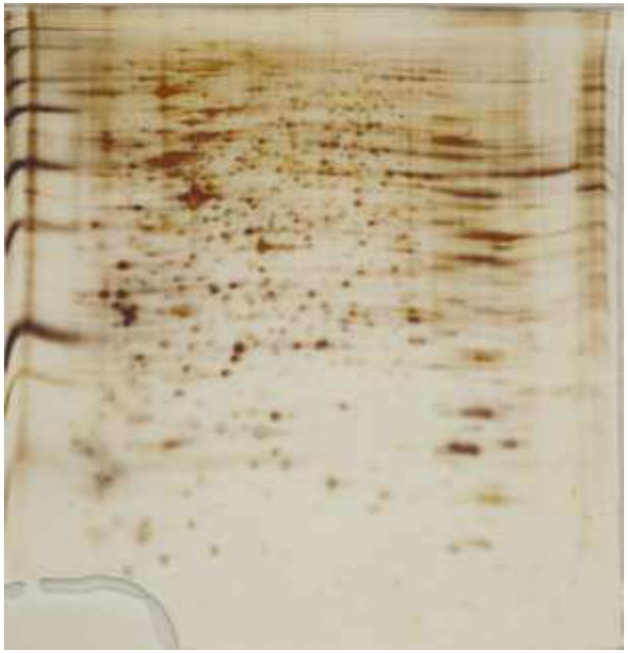

Jur kd DMSO

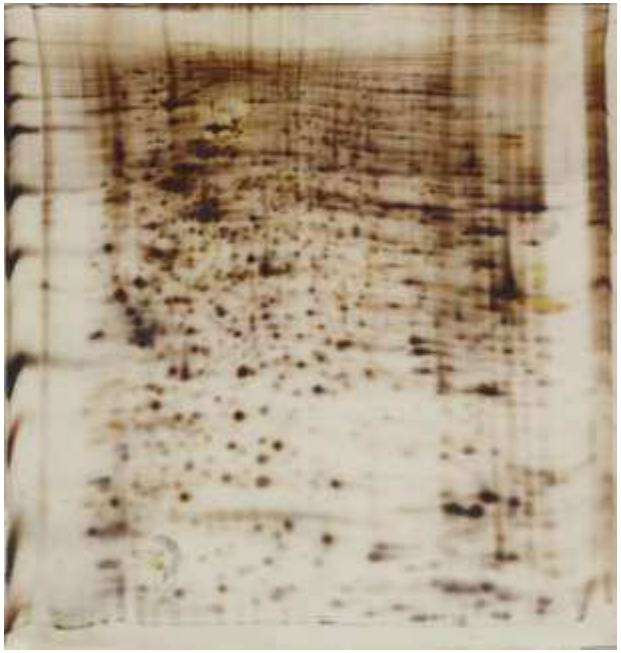

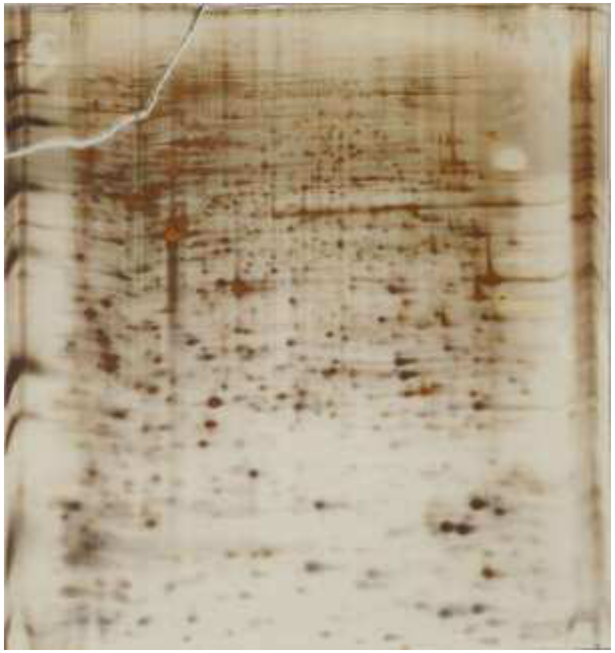

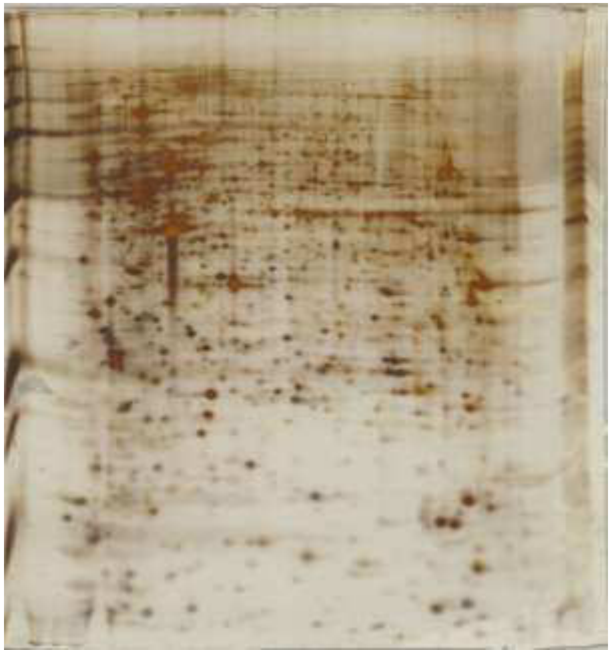

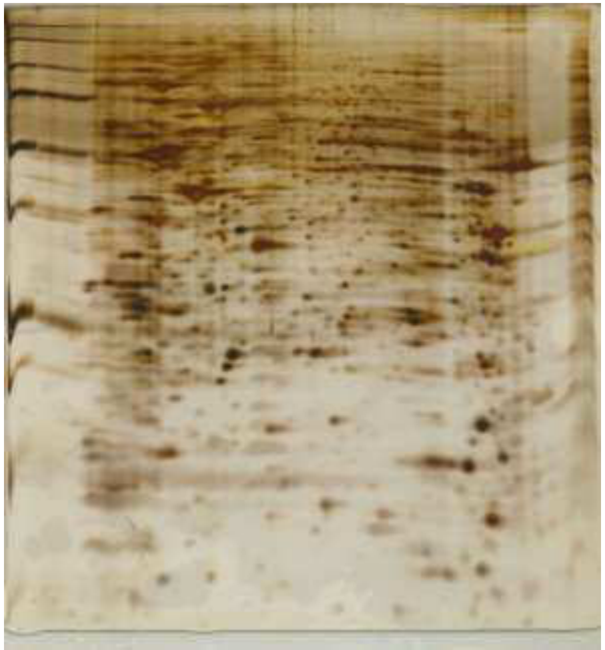

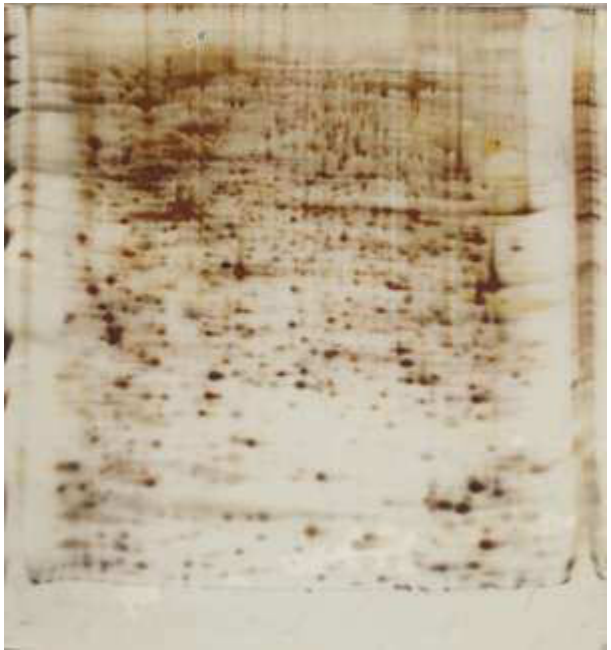

Jur kd 6-MP

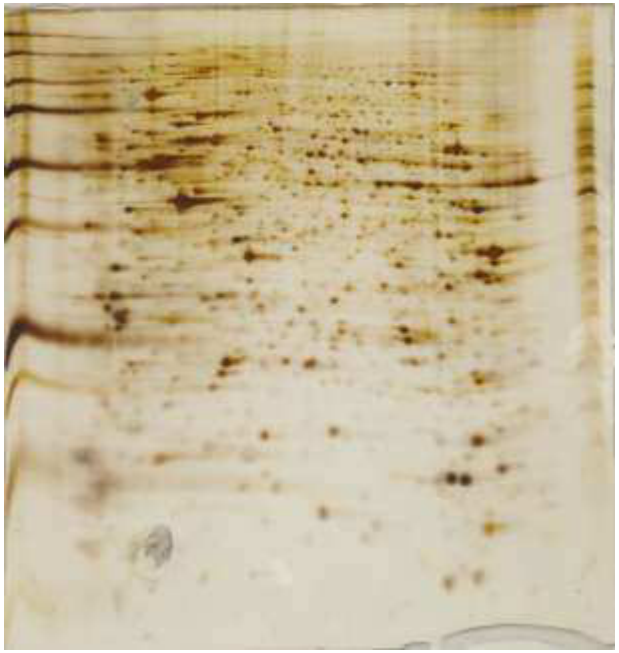

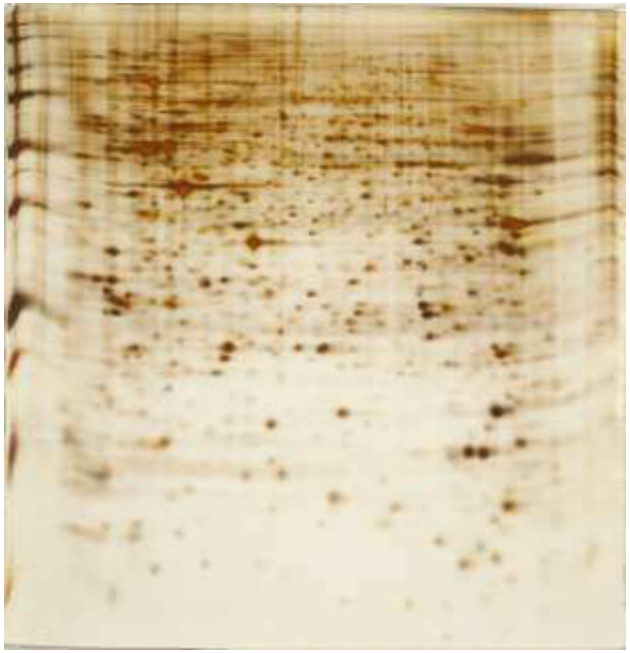

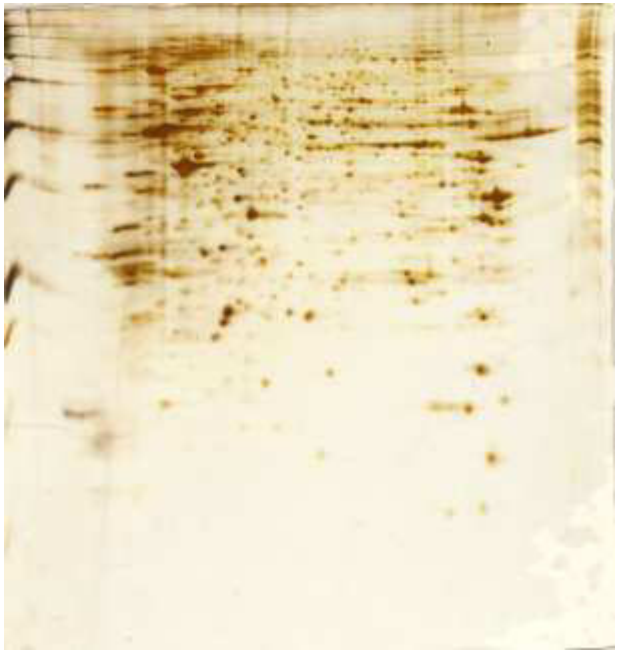

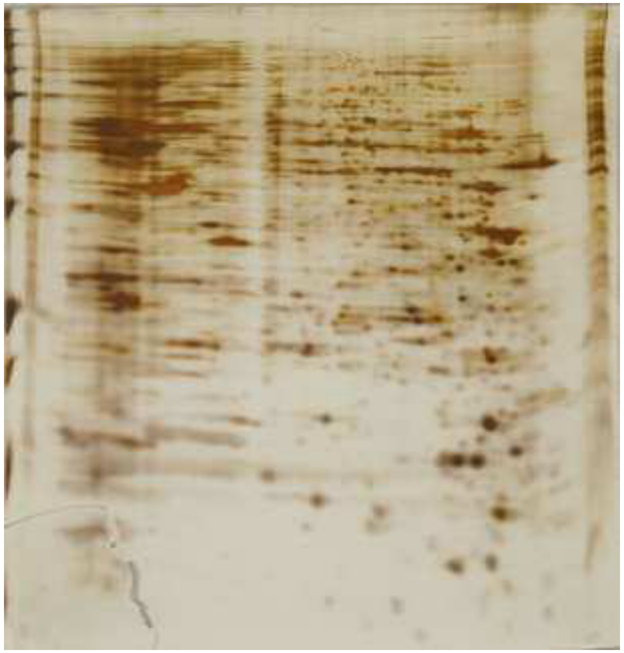

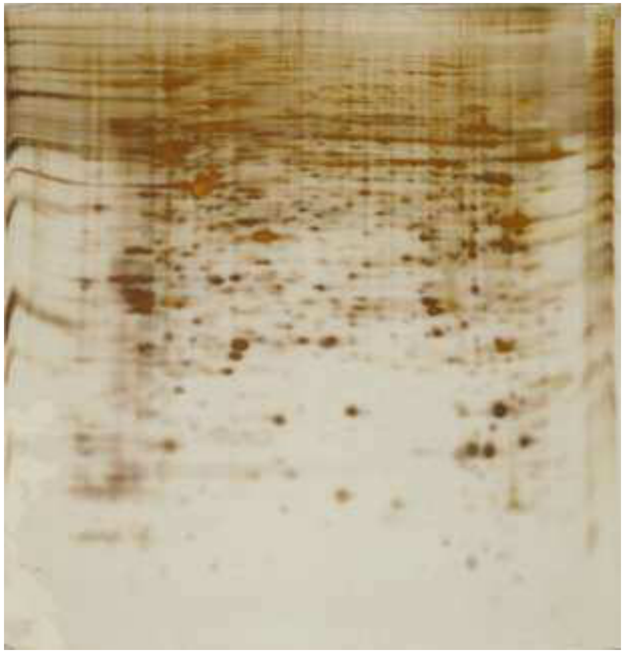

Jur kd 6-TG

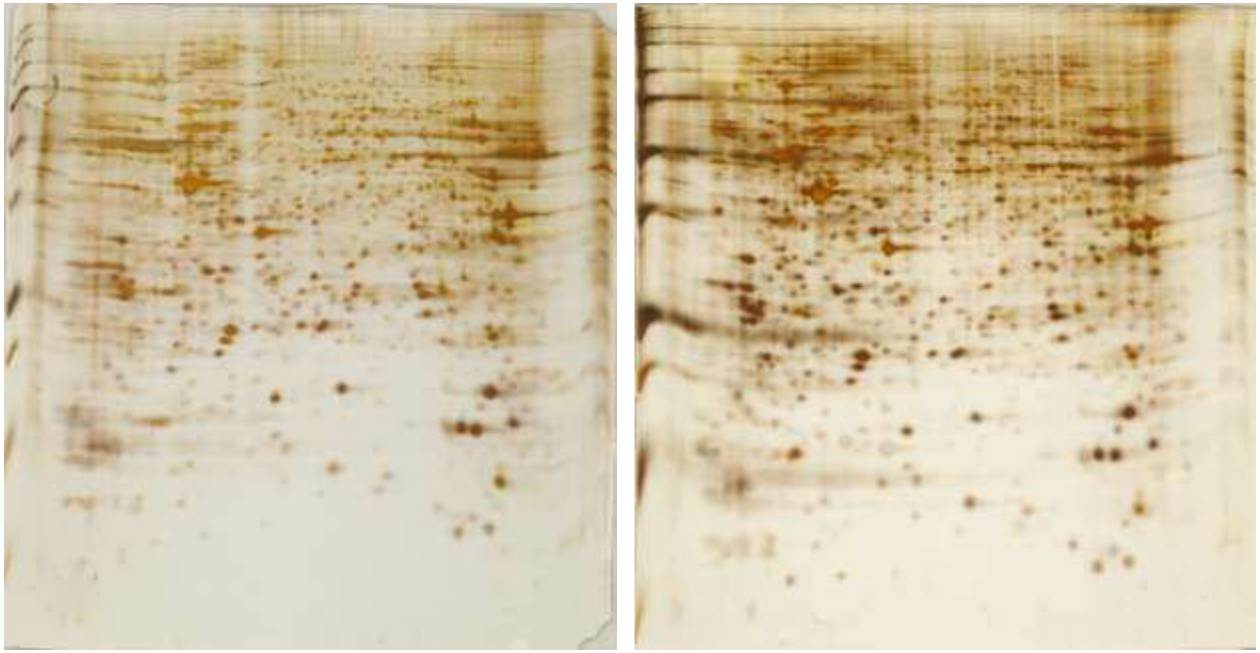

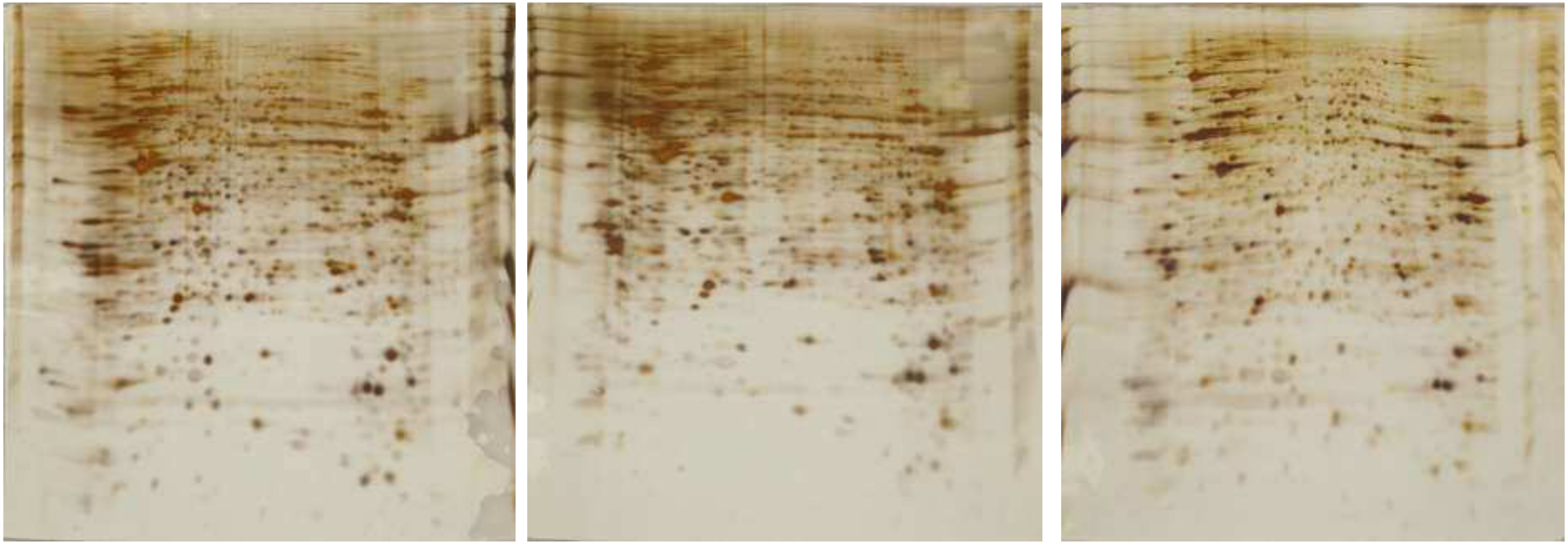

Suppl. Figure 1(b)

Jur wt DMSO

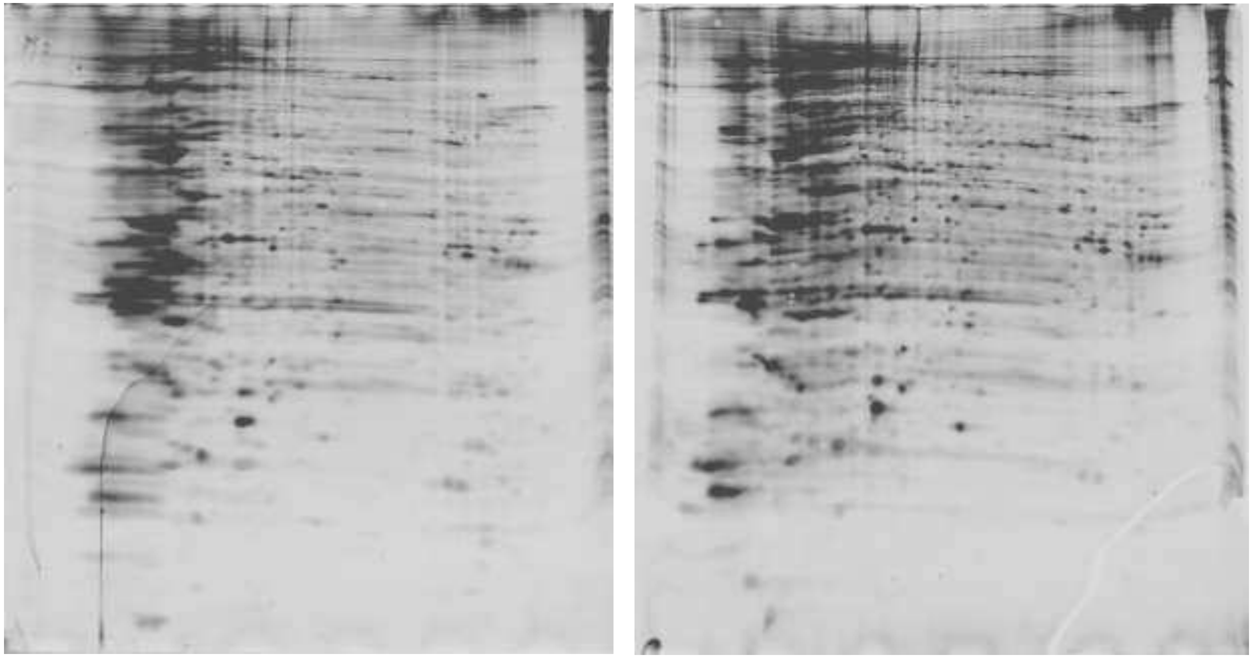

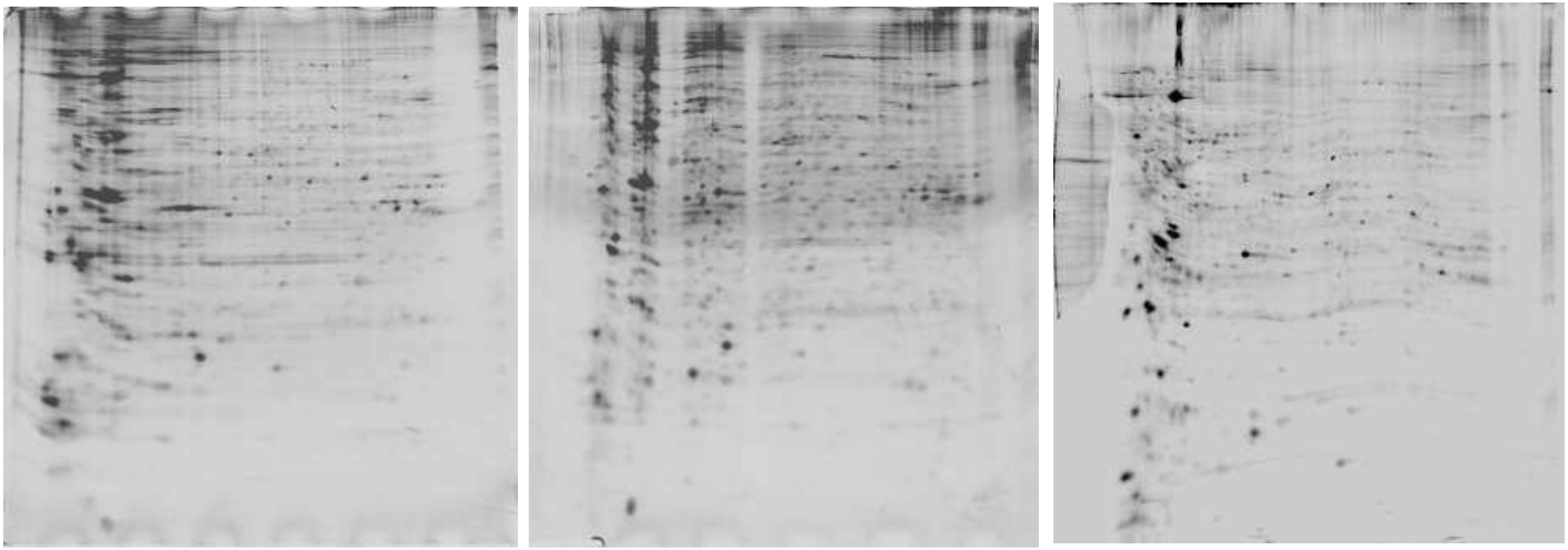

Jur wt 6-MP

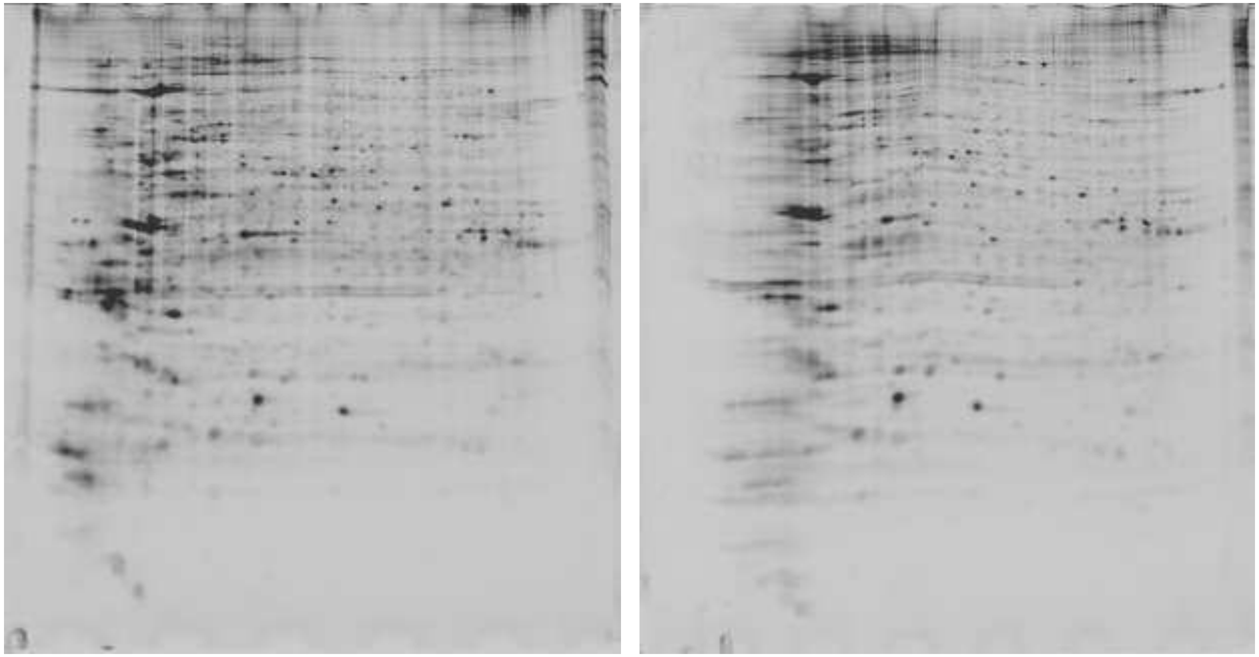

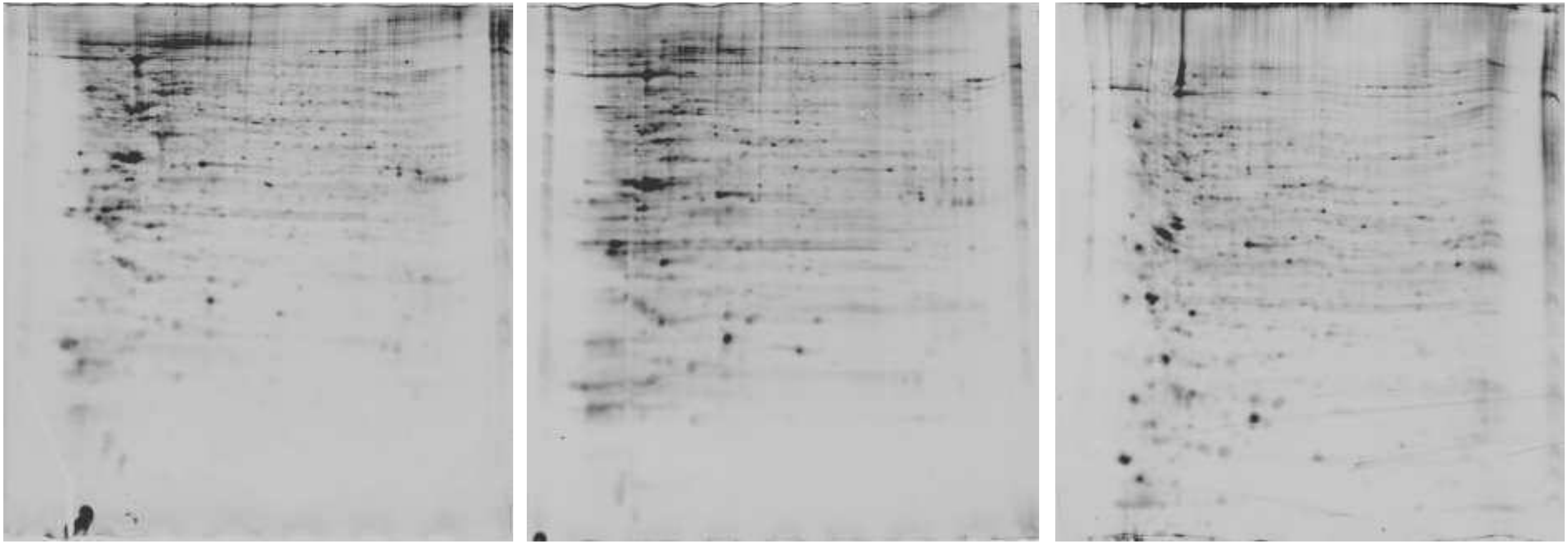

Jur wt 6-TG

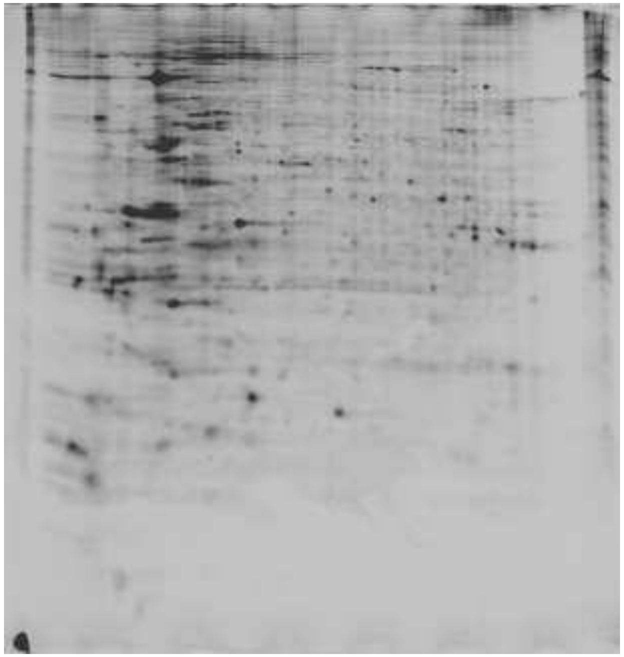

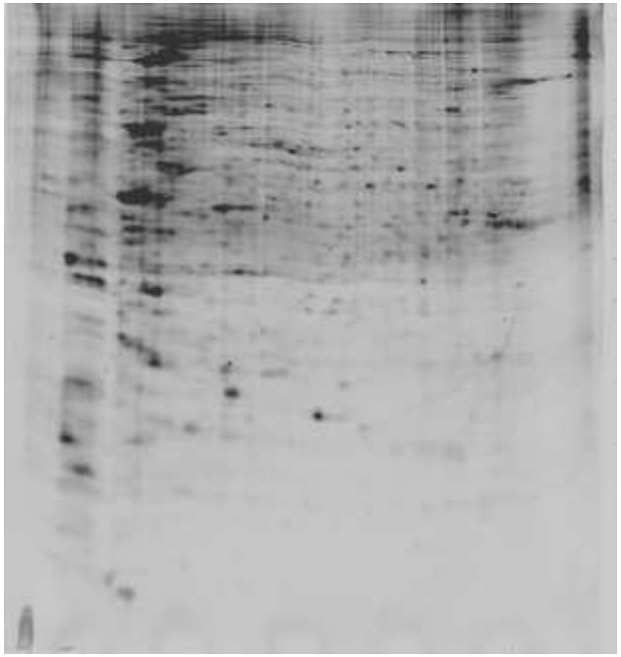

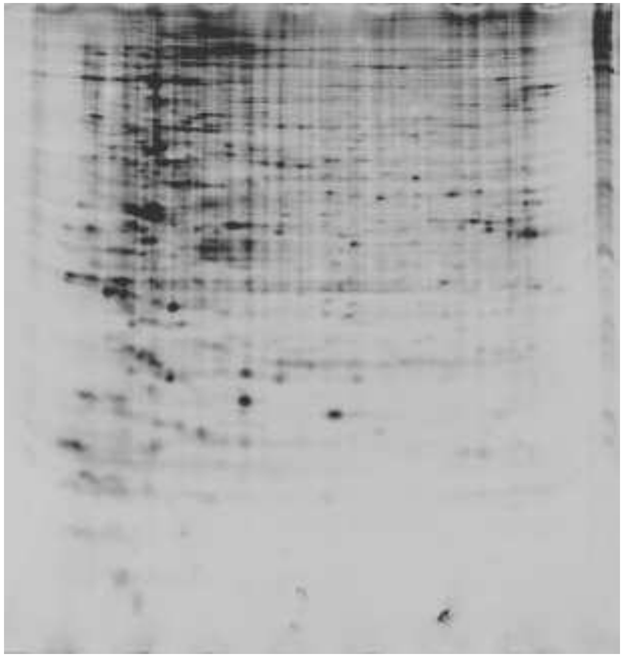

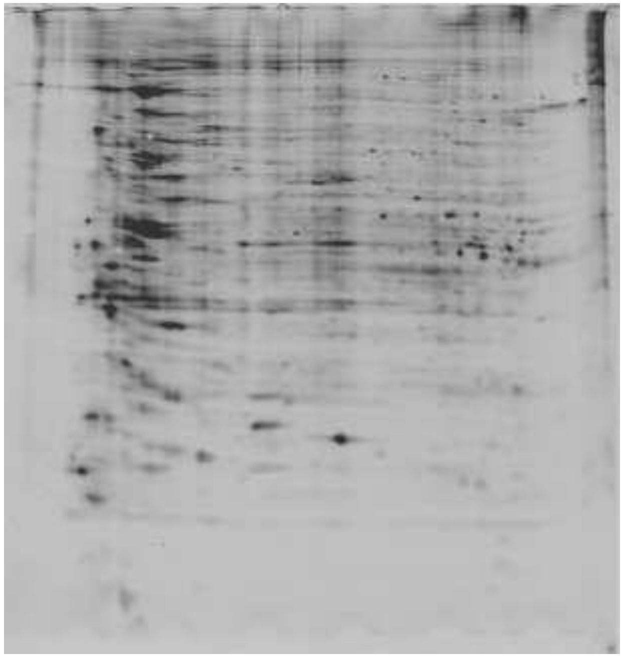

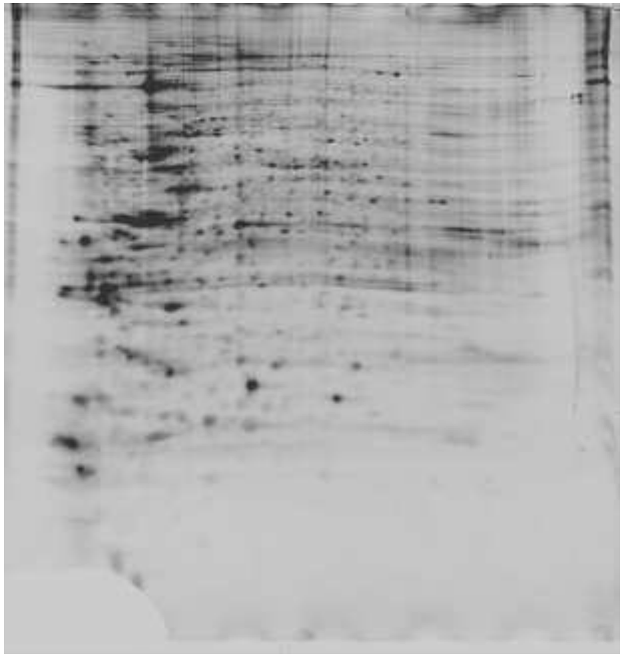

Jur kd DMSO

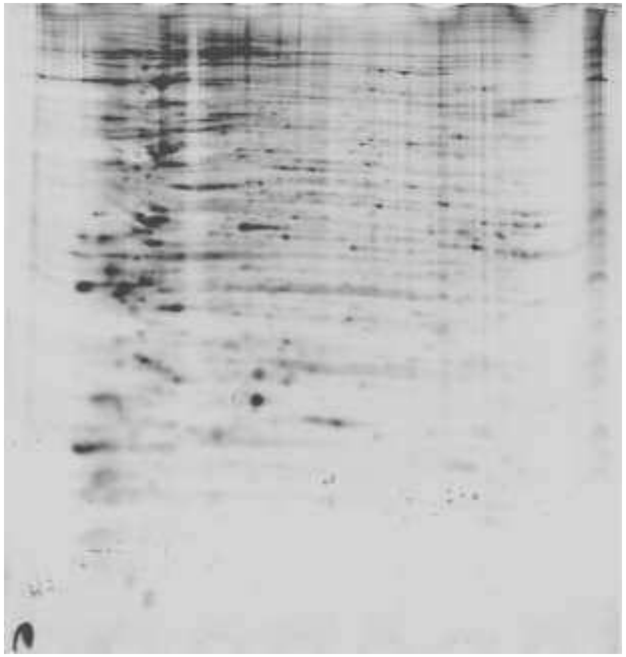

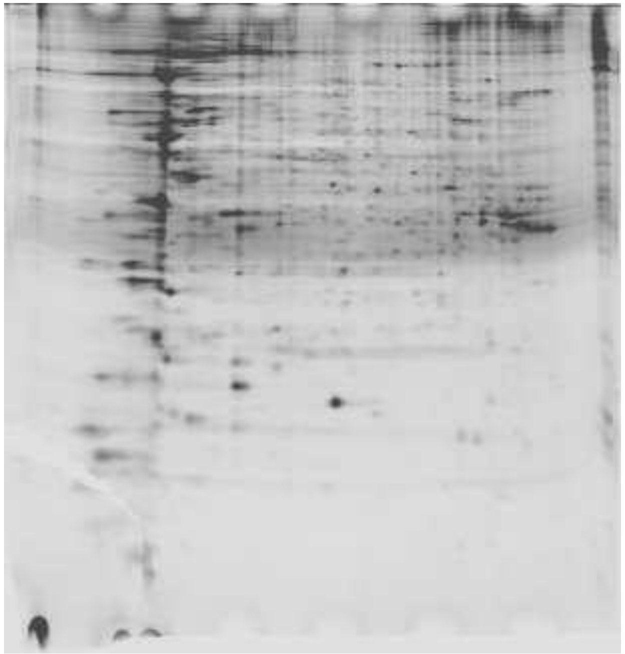

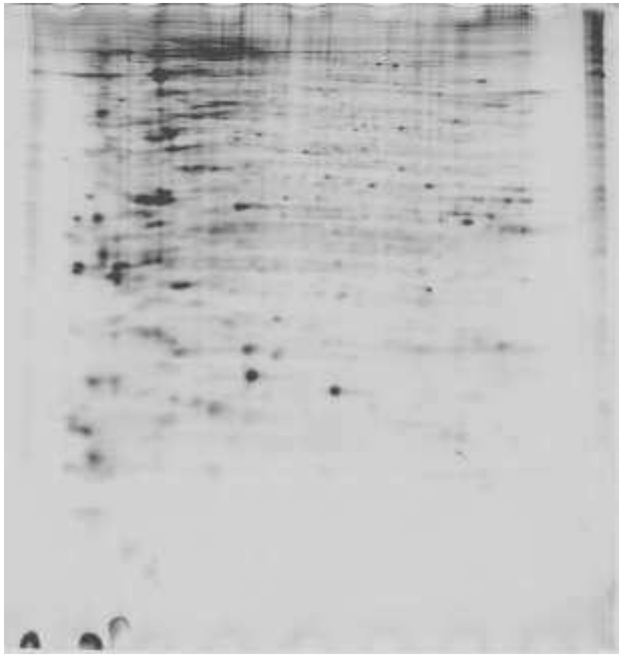

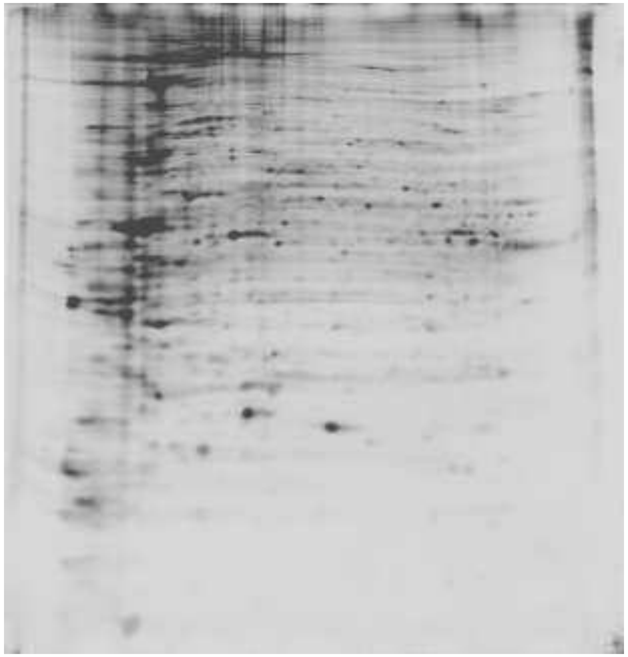

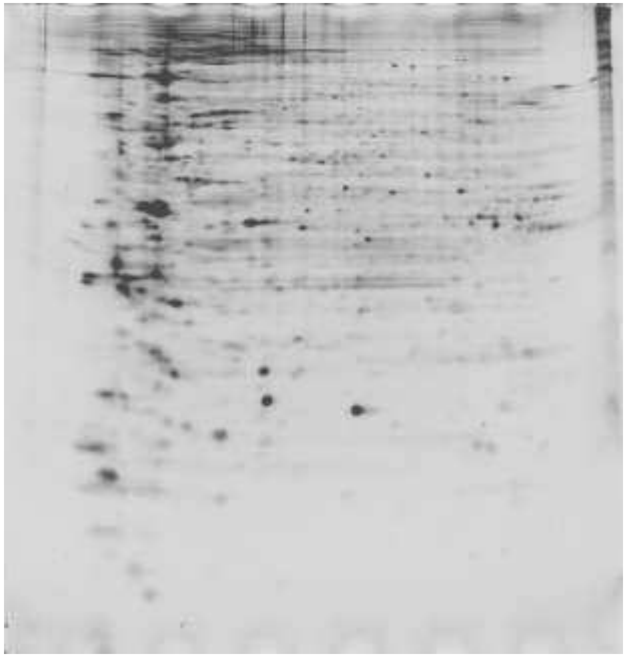

Jur kd 6-MP

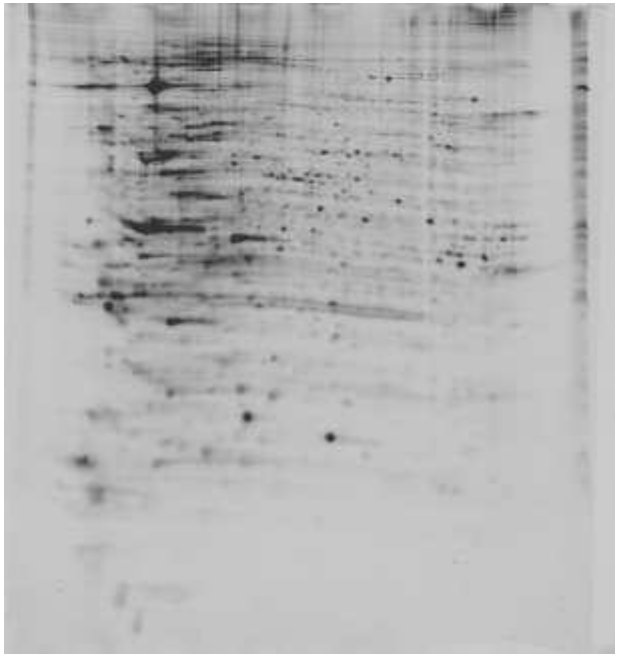

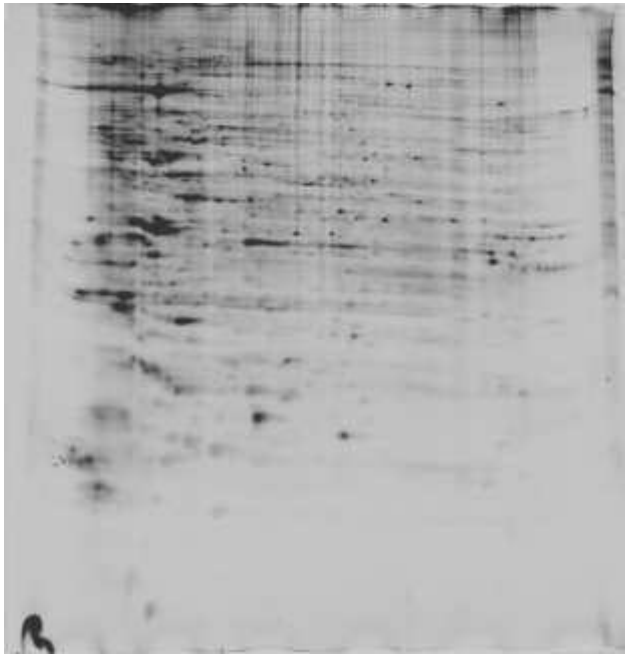

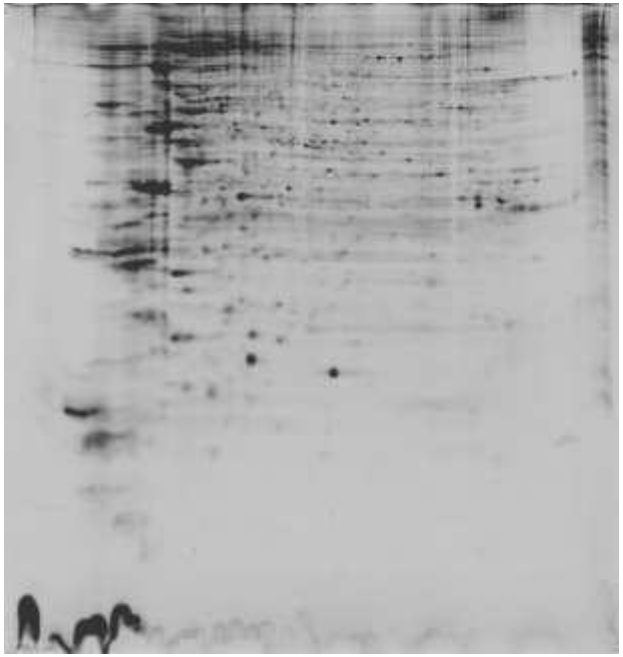

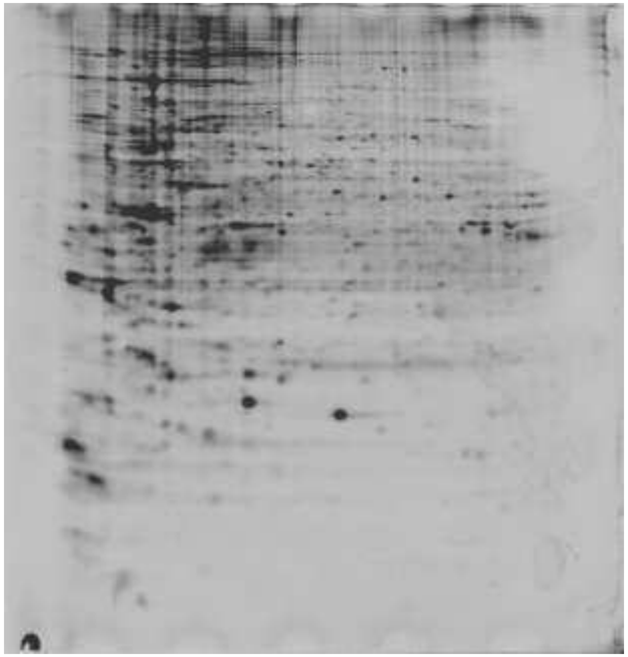

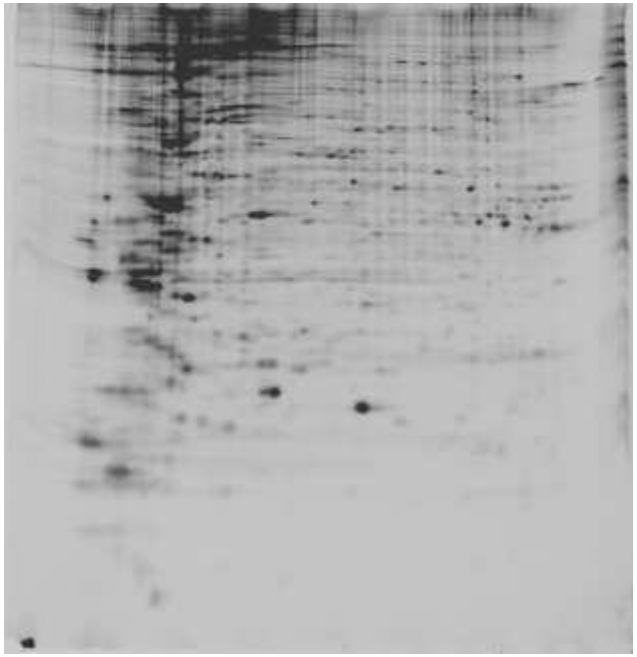

Jur kd 6-TG

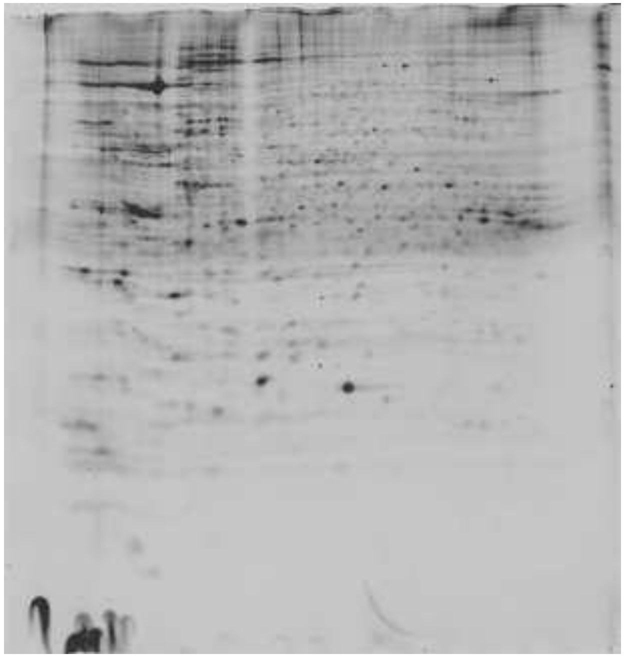

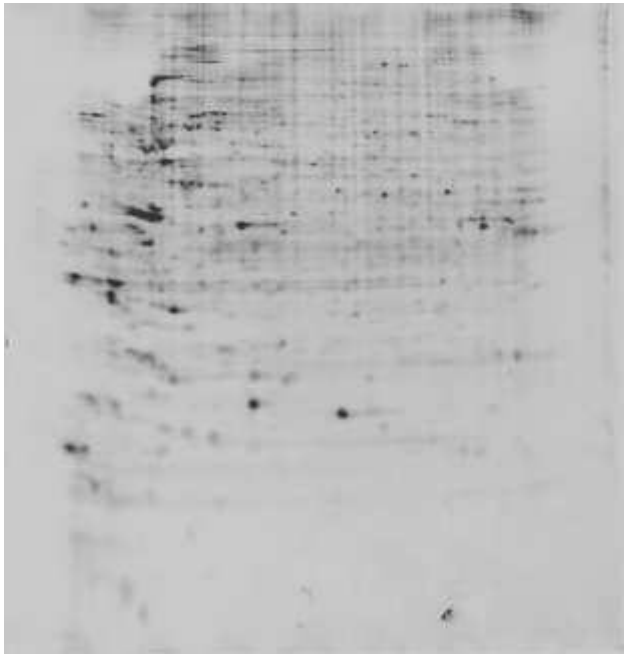

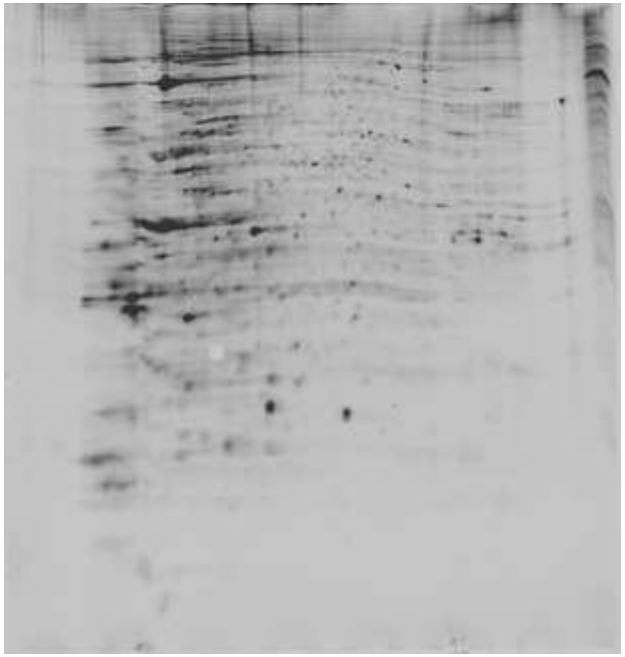

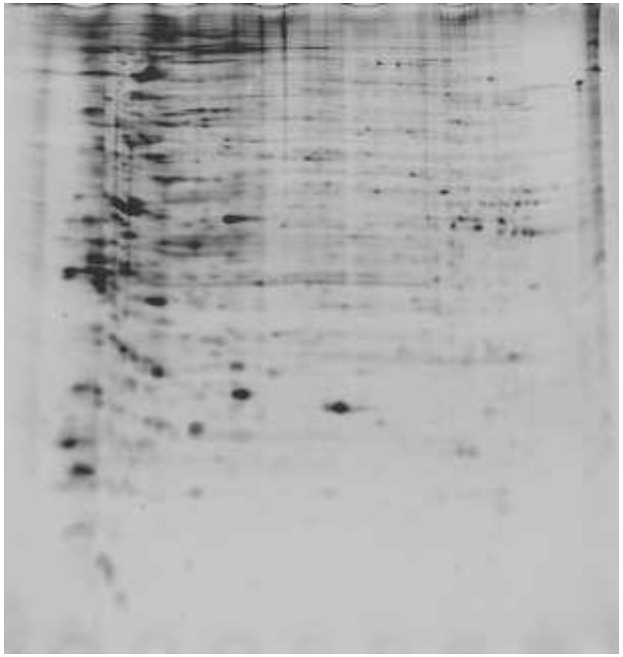

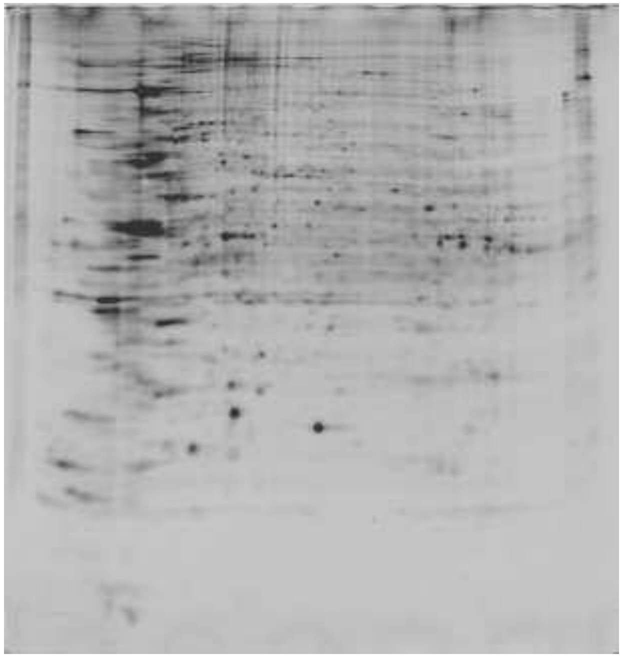

Suppl. Figure 2(a)

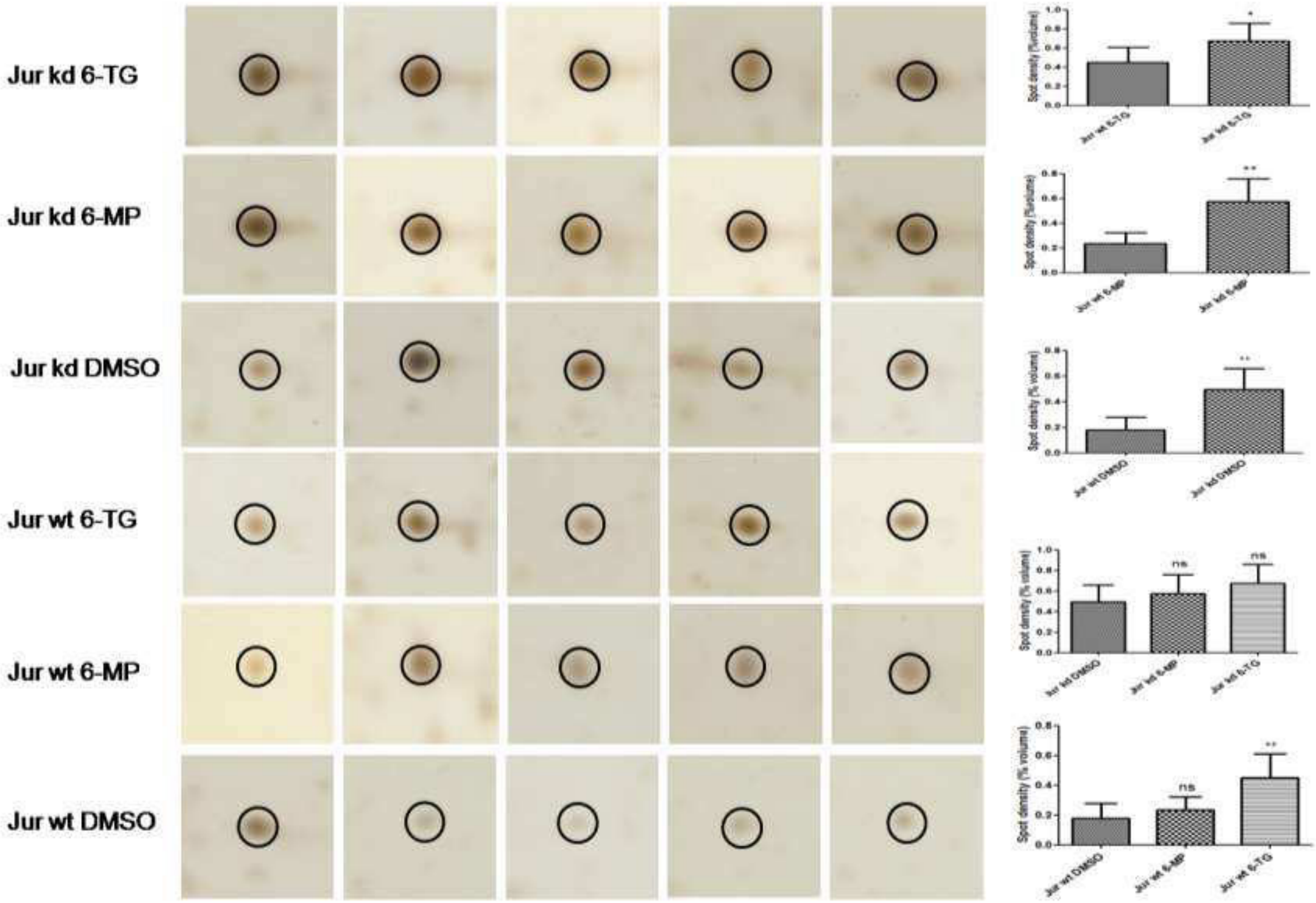

Suppl. Figure 2(b)

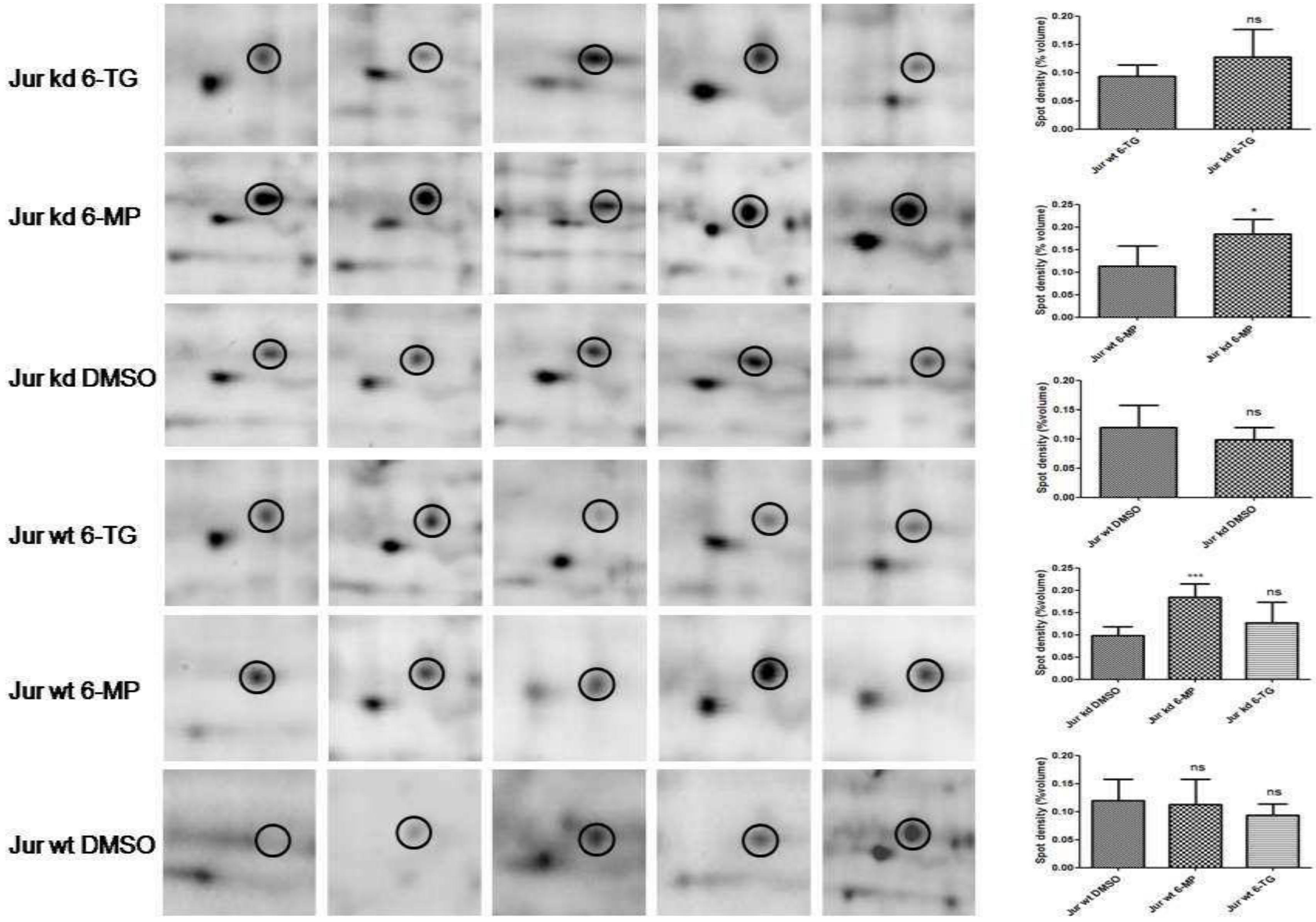

Suppl. Figure 3

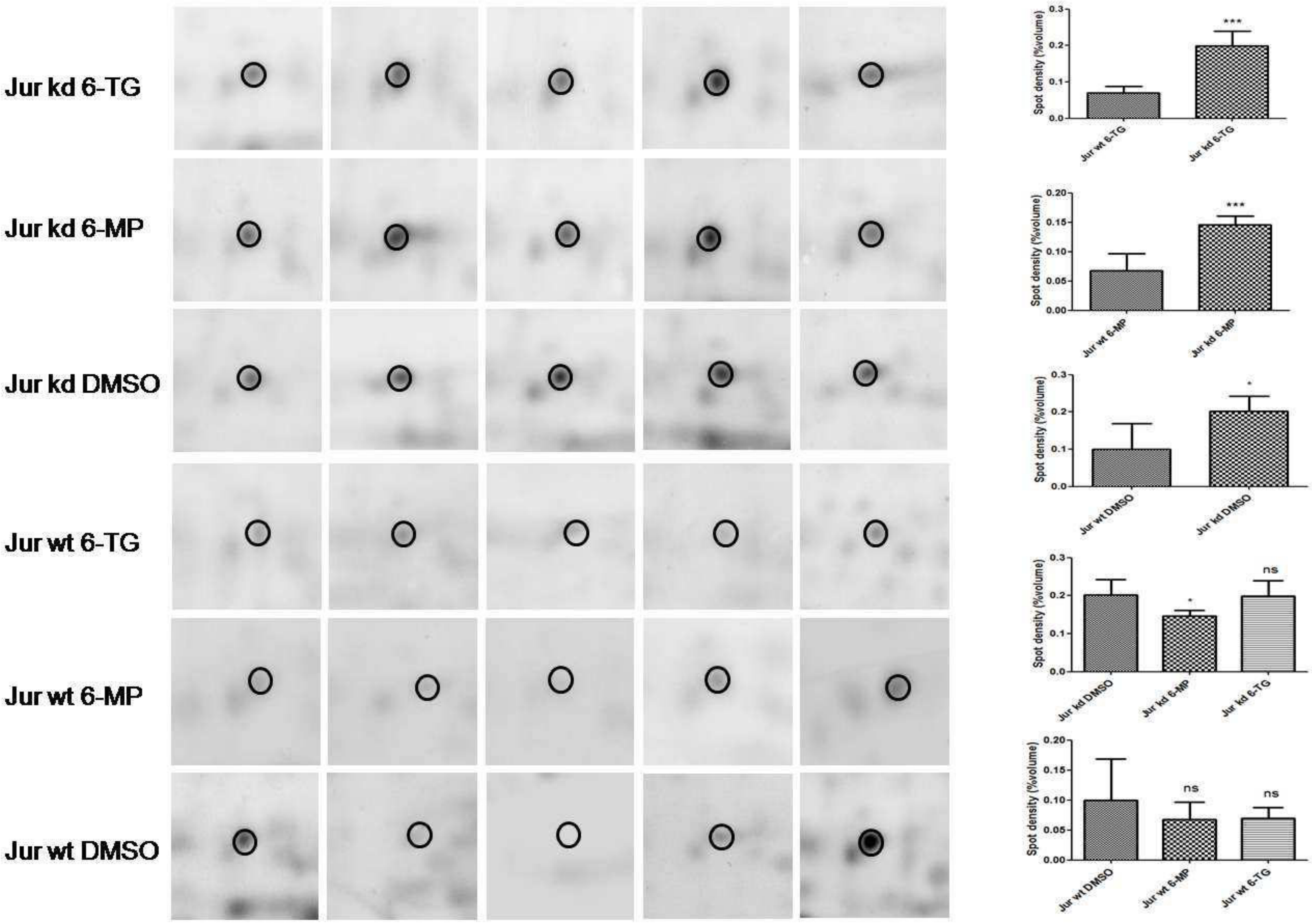

Suppl. Figure 4(a)

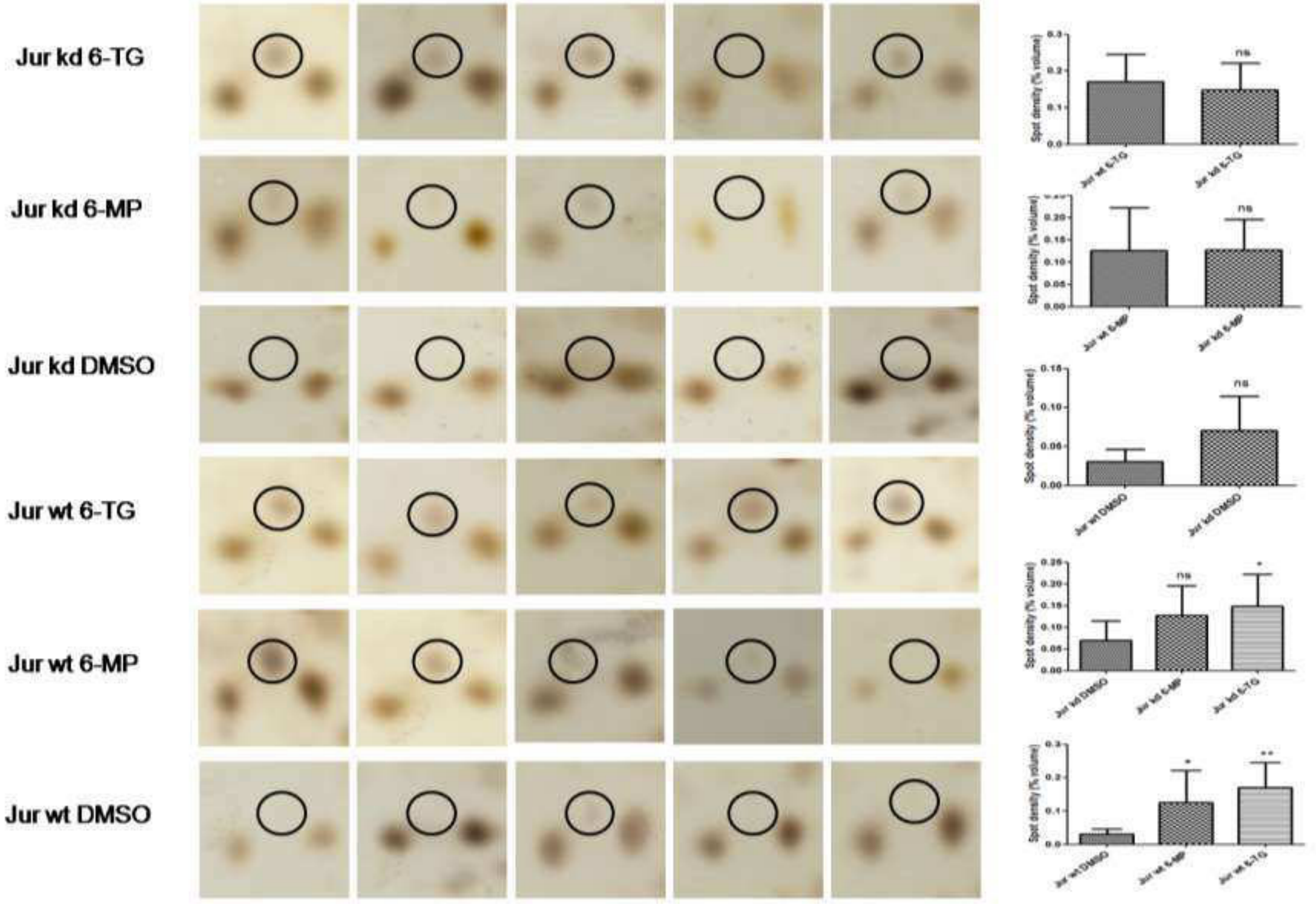

Suppl. Figure 4(b)

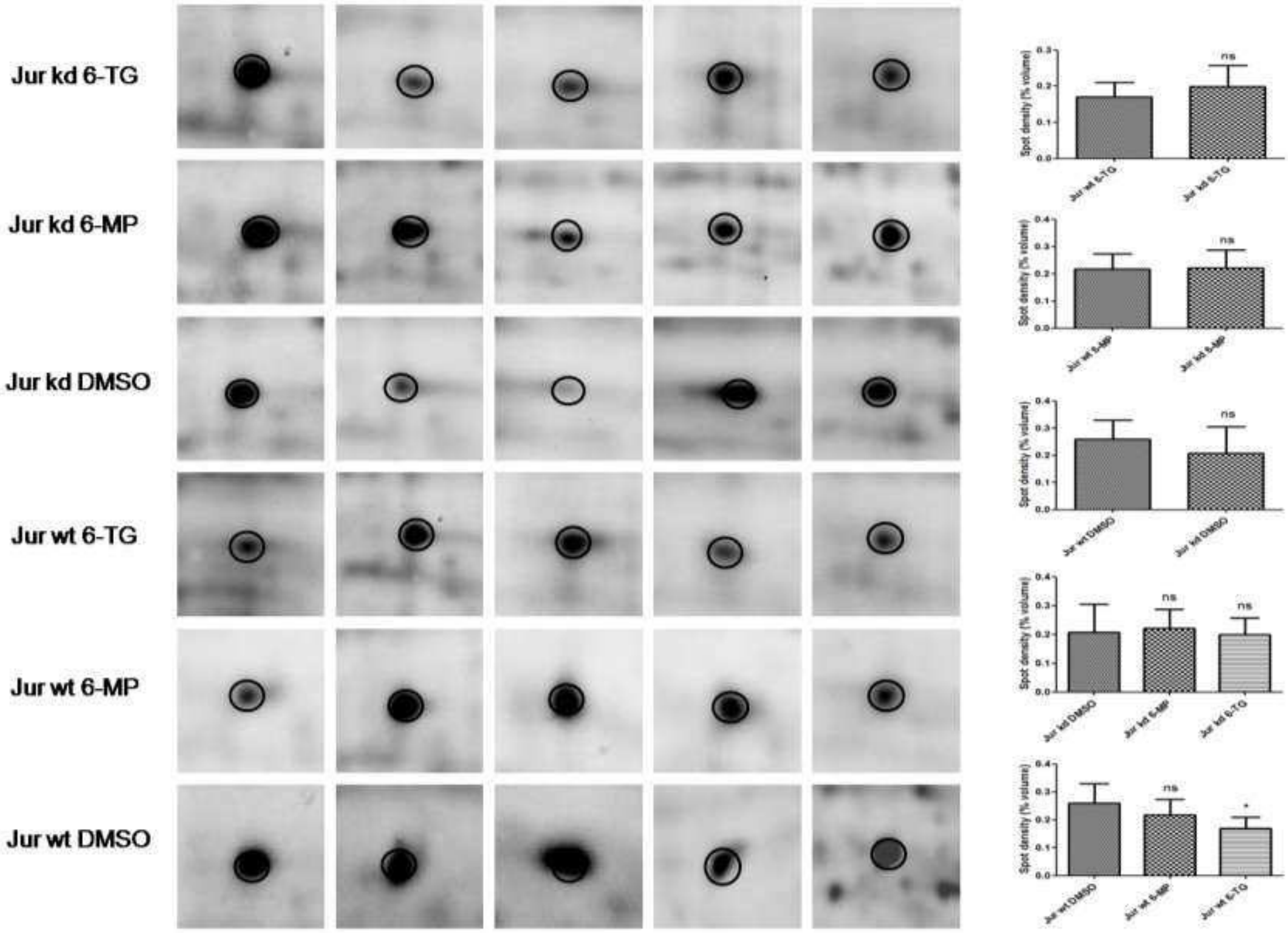

Suppl. Figure 5(a)

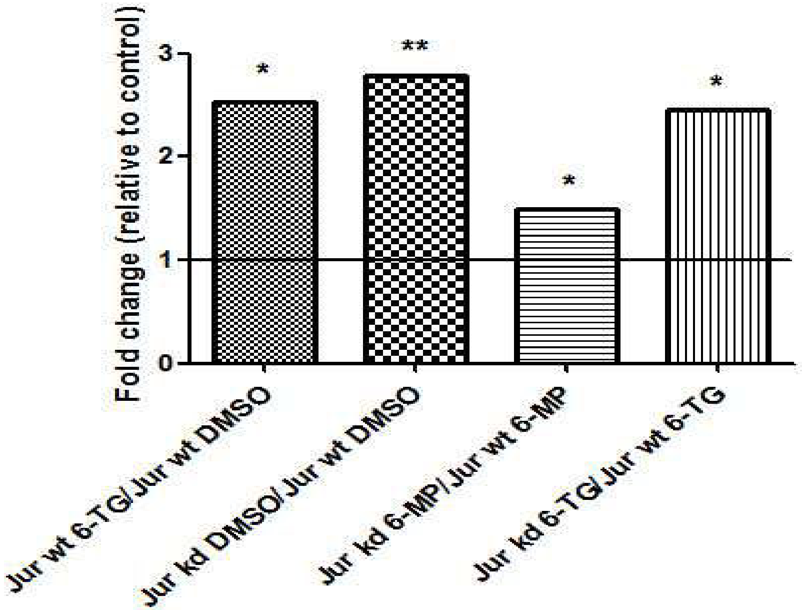

(b)

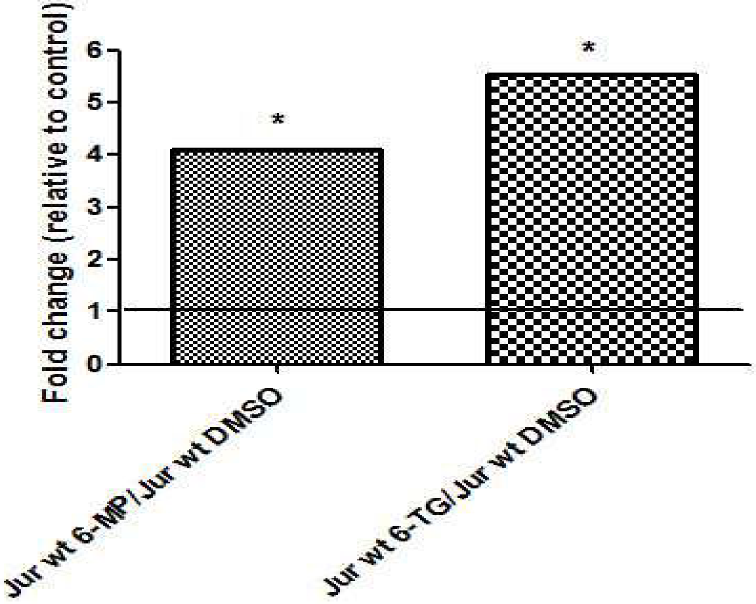

(c)

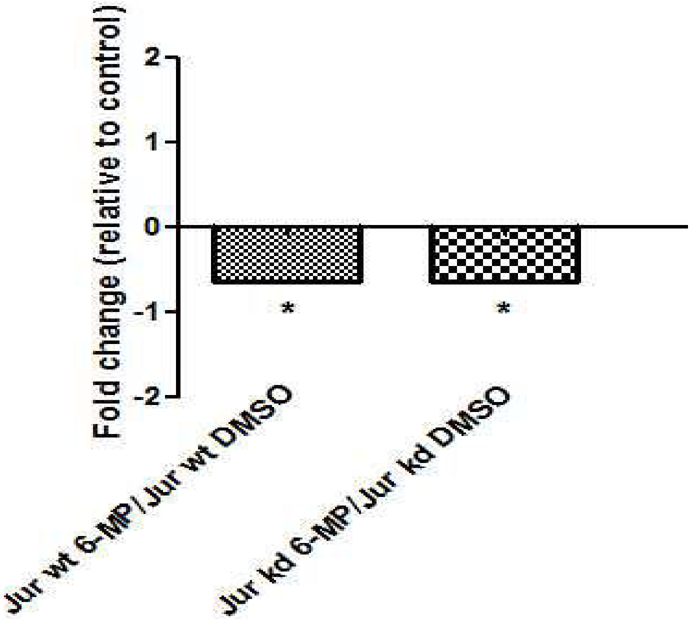

Suppl. Figure 6(a)

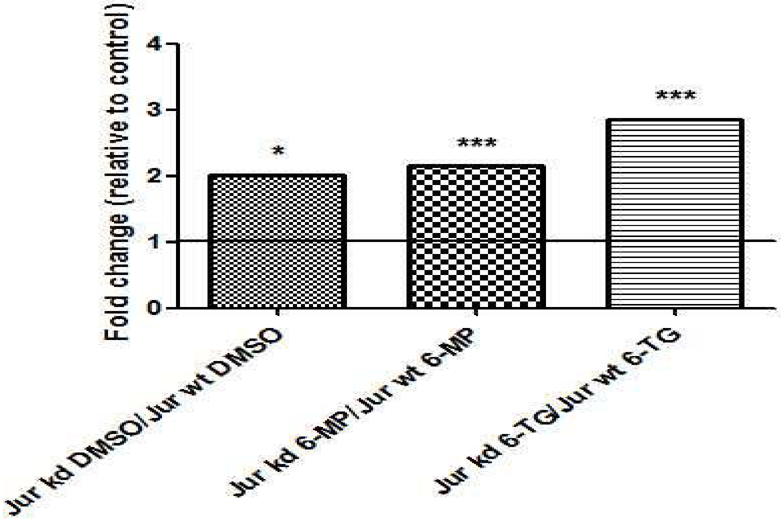

(b)

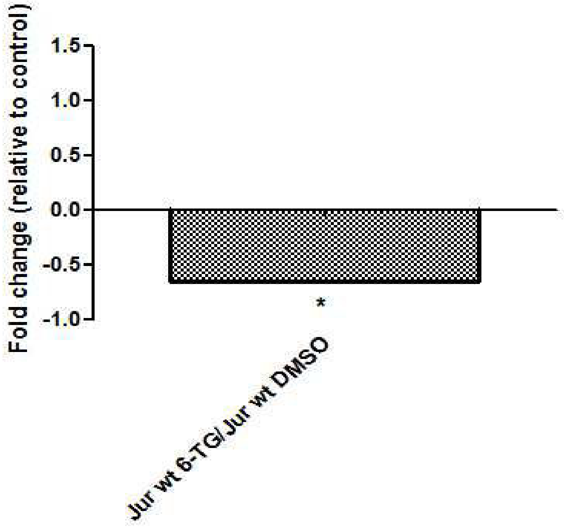

(c)

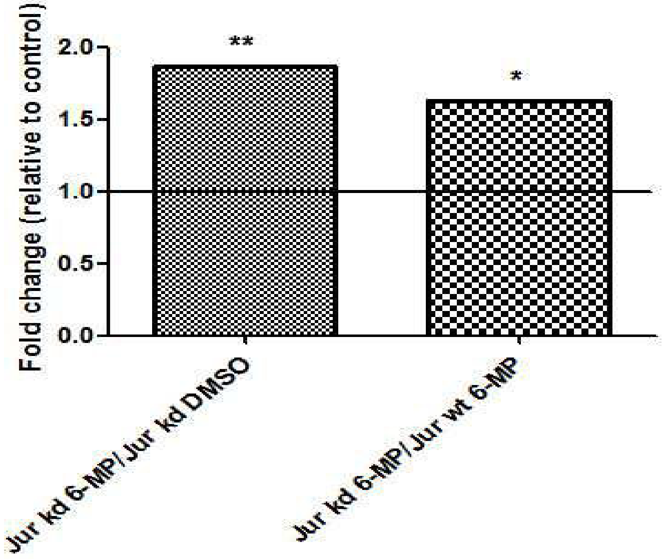

Table S I: MS/MS analysis table for differentially regulated proteins identified by Q-TOF MS/MS analysis

Footnotes: [1]Mass 1 = Molecular mass of the protein observed in Mascot search. [2]Mascot score 2 = >40 indicate identification or extensive homology ($p < 0.05$). [3]Peptide matched 3= Number of peptides matched with protein in MS/MS query. [4]pI 4 = Isoelectric point of the protein observed in Mascot search.

Spot no.	Acc. no.	Mass[1] (kDa)	pI[4]	Mascot Score[2]	Peptide matched[3]	Sequence Coverage	Protein name	MS/MS Analysis
1	Q9UBB4	53,5	5,12	82	3	4%	ATX10	1 MAAPRPPPAR LSGVMVPAPI QDLEALRALT ALFKEQRNRE TAPRTIFQRV<br>51 LDILKKSSHA VELACRDPSQ VENLASSLQL ITECFRCLRN ACIECSVNQN<br>101 SIRNLDTIGV AVDLILLFRE LR**VEQESLLT AFR**CGLQFLG NIASRNEDSQ<br>151 SIVWVHAFPE LFLSCLNHPD KKIVAYSSMI LFTSLNHERM KELEENLNIA<br>201 IDVIDAYQKH PESEWPFLII TDLFLKSPEL VQAMFPKLNN QERVTLLDLM<br>251 IAKITSDEPL TKDDIPVFLR HAELIASTFV DQCKTVLKLA SEEPPDDEEA<br>301 LATIRLLDVL CEMTVNTELL GYLQVFPGLL ERVIDLLRVI HVAGKETTNI<br>351 FSNCGCVRAE GDISNVANGF KSHLIRLIGN LCYKNKDNQD KVNELDGIPL<br>401 ILDNCNISDS NPFLTQWVIY AIR**NLTEDNS QNQDLIAKME EQGLADASLL**<br>451 **K**KVGFEVEKK GEKLILKSTR DTPKP<br>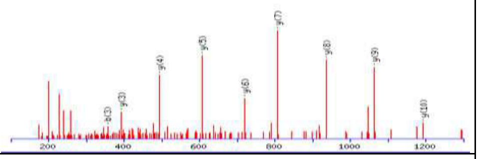
2	Q9UFU4	36,9	6,37	304	9	17%	HNRH3	**1** MDWVMK**HNGP NDASDGTVR**L RGLPFGCSKE EIVQFFQGLE IVPNGITLTM<br>**51** DYQGR**STGEA FVQFASK**EIA ENALGKHKER IGHR**YIEIFR** SSRSEIKGFY<br>**101** DPPRRLLGQR PGPYDRPIGG RGGYYGAGRG SMYDRMRRGG DGYDGGYGGF<br>**151** DDYGGYNNYG YGNDGFDDRM RDGRGMGGHG YGGAGDASSG FHGGHFVHMR<br>**201** GLPFR**ATEND IANFFSPLNP IR**VHIDIGAD GRATGEADVE FVTHEDAVAA<br>**251** MSKDKNNMQH RYIELFLNST PGGGSGMGGS GMGGYGR**DGM DNQGGYGSVG**<br>**301** **R**MGMGNNYSG GYGTPDGLGG YGRGGGGSGG YYGQGGMSGG GWRGMY<br>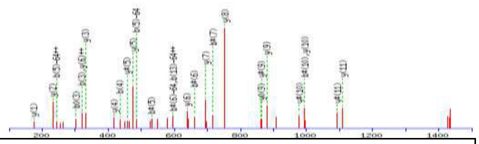
3	Q13011	35,8	8,16	174	6	14%	ECH1	**1** MAAGIVASRR LRDLLTRRLT GSNYPGLSIS LRLTGSSAQE EASGVALGEA<br>**51** PDHSYESLRV TSAQKHVLHV QLNRPNKRNA MNKVFWREMV ECFNKISRDA<br>**101** DCRAVVISGA GKMFTAGIDL MDMASDILQP KGDDVARISW YLRDIITRYQ<br>**151** ETFNVIERCP KPVIAAVHGG CIGGGVDLVT ACDIR**YCAQD AFFQVK**EVDV<br>**201** GLAADVGTLQ RLPK**VIGNQS LVNELAFTAR** KMMADEALGS GLVSRVFPDK<br>**251** EVMLDAALAL AAEISSKSPV AVQSTK**VNLL YSR**DHSVAES LNYVASWNMS<br>**301** MLQTQDLVK**S VQATTENKEL K**TVTFSKL<br>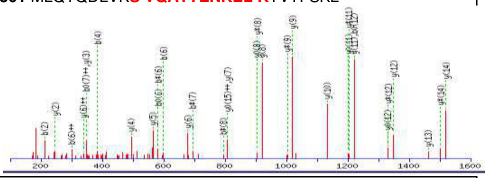
4	Q96Q81	26,7	9,65	111	4	12%	DUT	**1** MTPLCPRPAL CYHFLTSLLR SAMQNARGTA EGRSRGTLRA RPAPRPPAAQ<br>**51** HGIPRPLSSA GRLSQGCRGA STVGAAGWKG

								ELPKAGGSPA PGPETPAISP<br>**101** SKRARPAEVG GMQLRFAR**LS EHATAPTR**GS ARAAGYDLYS AYDYTIPPME<br>**151** KAVVK**TDIQI ALPSGCYGR**V APRSGLAAKH FIDVGAGVID EDYRGNVGVV<br>**201** LFNFGKEKFE VKKGDR**IAQL ICER**IFYPEI EEVQALDDTE RGSGGFGSTG<br>**251** KN<br>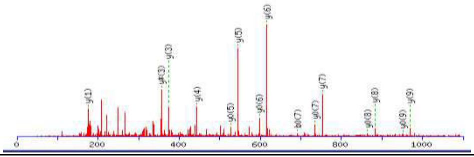
5	P23528	18,5	8,22	110	3	16%	COF1	**1** MASGVAVSDG VIKVFNDMKV RKSSTPEEVK KRKKAVLFCL SEDKKNIILE<br>**51** EGKEILVGDV GQTVDDPYAT FVKMLPDKDC R**YALYDATYE TK**ESKKEDLV<br>**101** FIFWAPESAP LKSKMIYASS KDAIKKKLTG IKHELQANCY EEVKDRCTLA<br>**151** EKLGGSAVIS LEGKPL<br>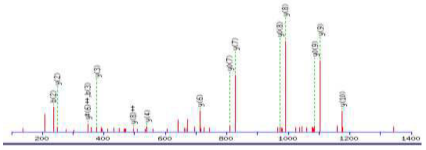
6	P16949	17,3	5,76	181	5	29%	STMN1	**1** MASSDIQVKE LEKR**ASGQAF ELILSPR**SKE SVPEFPLSPP KKK**DLSLEEI**<br>**51** **QK**KLEAAEER RKSHEAEVLK QLAEKREHEK EVLQK**AIEEN NNFSK**MAEEK<br>**101** LTHKMEANKE NREAQMAAKL ERLREKDKHI EEVRKNK**ESK DPADETEAD**<br>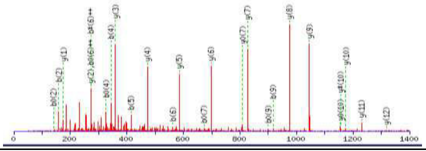
7	Q9P1F3	9	5,86	43	2	27%	ABRAL	**1** MNVDHEVNLL VEEIHRLGSK NADGKLSVK**F GVLFRDDKCA NLFEALVGTL**<br>51 **K**AAKRRKIVT YPGELLLQGV HDDVDIILLQ D<br>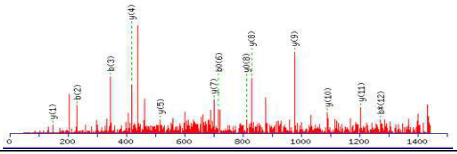
8	P10599	11,7	4,82	244	10	40%	THIO	**1** MVKQIESK**TA FQEALDAAGD K**LVVVDFSAT WCGPCKMIKP FFHSLSEKYS<br>**51** NVIFLEVDVD DCQDVASECE VK**CMPTFQFF K**KGQK**VGEFS GANKEKLEAT**<br>**101** **INELV**<br>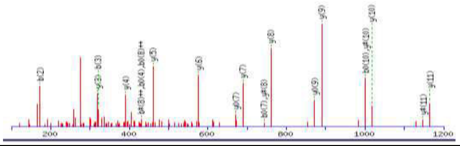
9	P07737	15	8,44	113	4	22%	PROF1	**1** MAGWNAYIDN LMADGTCQDA AIVGYKDSPS VWAAVPGK**TF VNITPAEVGV**<br>**51** **LVGKDR**SSFY VNGLTLGGQK CSVIRDSLLQ DGEFSMDLRT K**STGGAPTFN**<br>**101** **VTVTK**TDKTL VLLMGKEGVH GGLINKKCYE MASHLRRSQY<br>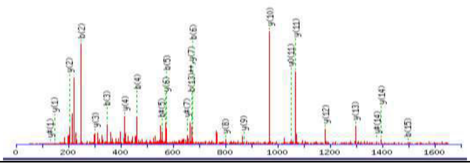
10	P14174	12,5	4,74	175	4	17%	MIF	**1** M**PMFIVNTNV PR**ASVPDGFL SELTQQLAQA TGKPPQYIAV HVVPDQLMAF<br>**51** GGSSEPCALC SLHSIGKIGG AQNRSYSK**LL CGLLAER**LRI SPDRVYINYY<br>**101** DMNAANVGWN NSTFA

								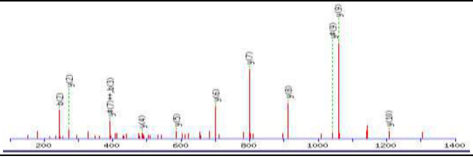
11	P69905	15,2	8,72	31	2	6%	HBA	1 MVLSPADKTN VKAAWGKVGA HAGEYGAEAL ER**MFLSFPTT K**TYFPHFDLS<br>51 HGSAQVKGHG KKVADALTNA VAHVDDMPNA LSALSDLHAH KLRVDPVNFK<br>101 LLSHCLLVTL AAHLPAEFTP AVHASLDKFL ASVSTVLTSK YR<br>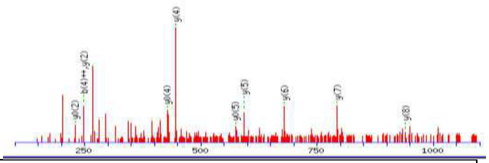
12	Q9BRA2	13,9	5,4	187	5	15%	TXD17	**1** MARYEEVSVS GFEEFHRAVE QHNGK**TIFAY FTGSK**DAGGK SWCPDCVQAE<br>**51** PVVREGLKHI SEGCVFIYCQ VGEKPYWKDP NNDFRKNLK**V TAVPTLLK**YG<br>**101** TPQKLVESEC LQANLVEMLF SED<br>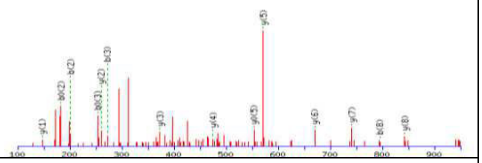
13	P58546	12,8	5,27	118	3	14%	MTPN	**1** MCDKEFMWAL KNGDLDEVKD YVAKGEDVNR TLEGGRKPLH YAADCGQLEI<br>**51** LEFLLLKGAD INAPDKHHIT PLLSAVYEGH VSCVKLLLSK GADKTVK**GPD**<br>**101** **GLTAFEATDN QAIK**ALLQ<br>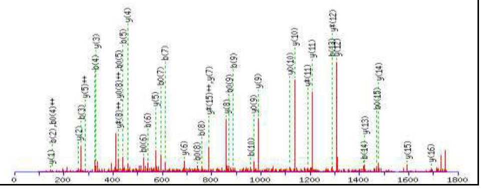

Table S II: MS/MS analysis table for differentially regulated phospho proteins identified by Q-TOF MS/MS analysis

Footnotes: [1]Mass 1 = Molecular mass of the protein observed in Mascot search. [2]Mascot score 2 = >40 indicate identification or extensive homology ($p < 0.05$). [3]Peptide matched 3= Number of peptides matched with protein in MS/MS query. [4]pI 4 = Isoelectric point of the protein observed in Mascot search.

Spot no.	Acc. no.	Mass[1] (kDa)	pI[4]	Mascot Score[2]	Peptide matched[3]	Sequence Coverage	Protein name	MS/MS Analysis
1	Q01813	85,5	7,50	184	11	11%	K6PP	1 MDADDSRAPK GSLRKFLEHL SGAGKAIGVL TSGGDAQGMN AAVRAVVRMG<br>51 IYVGAKVYFI YEGYQGMVDG GSNIAEADWE SVSSILQVGG TIIGSARCQA<br>101 FRTREGRLKA ACNLLQRGIT NLCVIGGDGS LTGANLFRK**E WSGLLEELAR**<br>151 NGQIDKEAVQ KYAYLNVVGM VGSIDNDFCG TDMTIGTDSA LHR**IIEVVDA**<br>201 **IMTTAQSHQR TFVLEVMGR**H CGYLALVSAL ACGADWVFLP ESPPEEGWEE<br>251 QMCVKLSENR ARKKRLNIII VAEGAIDTQN KPITSEKIK**E LVVTQLGYDT**<br>301 **R**VTILGHVQR GGTPSAFDRI LASRMGVEAV IALLEATPDT PACVVSLNGN<br>351 HAVRLPLMEC VQMTQDVQKA MDER**RFQDAV R**LRGRSFAGN LNTYKRLAIK<br>401 LPDDQIPKTN CNVAVINVGA PAAGMNAAVR SAVRVGIADG HR**MLAIYDGF**<br>451 **DGFAK**GQIKE IGWTDVGGWT GQGGSILGTK RVLPGKYLEE IATQMRTHSI<br>501 NALLIIGGFE AYLGLLELSA AREKHEEFCV PMVMVPATVS NNVPGSDFSI<br>551 GADTALNTIT DTCDRIKQSA SGTKRRVFII ETMGGYCGYL ANMGGLAAGA<br>601 DAAYIFEEPF DIR**DLQSNVE HLTEK**MKTTI QRGLVLRNES CSENYTTDFI<br>651 YQLYSEEGKG VFDCRKNVLG HMQQGGAPSP FDRNFGTKIS ARAMEWITAK<br>701 LKEARGRGKK **FTTDDSICVL GISKRNVIFQ PVAELKK**QTD FEHRIPKEQW<br>751 WLKLRPLMKI LAKYKASYDV SDSGQLEHVQ PWSV<br>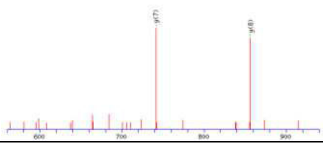
2	P40227	58	6,23	366	12	21%	TCPZ	1 MAAVKTLNPK AEVAR**AQAAL AVNISAARGL QDVLR**TNLGP KGTMK**MLVSG**<br>51 **AGDIK**LTKDG NVLLHEMQIQ HPTASLIAKV ATAQDDITGD GTTSNVLIIG<br>101 ELLKQADLYI SEGLHPR**IIT EGFEAAKEKA LQFLEEVKVS R**EMDRETLID<br>151 VARTSLRTKV HAELADVLTE AVVDSILAIK KQDEPIDLFM IEIMEMKHKS<br>201 ETDTSLIRGL VLDHGARHPD MKKRVEDAYI LTCNVSLEYE K**TEVNSGFFY**<br>251 **K**SAEEREKLV KAERKFIEDR VKKIIELKRK VCGDSDKGFV VINQKG**IDPF**<br>301 **SLDALSKEGI VALR**RAKRRN MERLTLACGG VALNSFDDLS PDCLGHAGLV<br>351 YEYTLGEEKF TFIEKCNNPR SVTLLIKGPN KHTLTQIKDA VRDGLRAVKN<br>401 AIDDGCVVPG AGAVEVAMAE ALIKHKPSVK GR**AQLGVQAF ADALLIIPKV**<br>451 **LAQNSGFDLQ ETLVK**IQAEH SESGQLVGVD LNTGEPMVAA EVGVWDNYCV<br>501 KKQLLHSCTV IATNILLVDE IMRAGMSSLK G<br>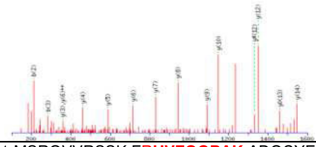
3	P31146	51	6,25	131	6	10%	COR1A	1 MSRQVVRSSK F**RHVFGQPAK** ADQCYEDVRV **SQTTWDSGFC AVNPKFVALI**<br>51 **CEASGGGAFL VLPLGK**TGRV DKNAPTVCGH TAPVLDIAWC PHNDNVIASG<br>101 SEDCTVMVWE IPDGGLMLPL REPVVTLEGH TKRVGIVAWH TTAQNVLLSA<br>151 GCDNVIMVWD VGTGAAMLTL GPEVHPDTIY SVDWSRDGGL ICTSCRDKRV<br>201 RIIEPRKGTV VAEKDRPHEG TRPVR**AVFVS EGKILTTGFS RMSERQVALW**<br>251 **DTK**HLEEPLS LQELDTSSGV LLPFFDPDTN IVYLCGKGDS SIRYFEITSE<br>301 APFLHYLSMF SSKESQRGMG YMPKRGLEVN KCEIARFYKL

								HERRCEPIAM<br>351 TVPRKSDLFQ EDLYPPTAGP DPALTAEEWL GGR**DAGPLLI SLKDGYVPPK**<br>401 SRELRVNRGL DTGRRRAAPE ASGTPSSDAV SRLEEEMRKL QATVQELQKR<br>451 LDRLEETVQA K<br>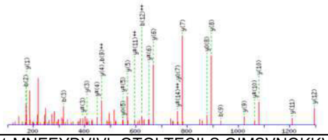
4	P50395	50,6	6,11	378	11	30%	GDIB	1 MNEEYDVIVL GTGLTECILS GIMSVNGKKV LHMDR**NPYYG GESASITPLE**<br>51 **DLYK**RFKIPG SPPESMGR**GR DWNVDLIPK**F LMANGQLVK**M LLYTEVTR**YL<br>101 DFK**VTEGSFV YKGGK**IYKVP STEAEALASS LMGLFEKRRF RK**FLVYVANF**<br>151 **DEKDPRTFEG IDPKK**TTMRD VYKKFDLGQD VIDFTGHALA LYR**TDDYLDQ**<br>201 **PCYETINRIK LYSESLAR**YG KSPYLYPLYG LGELPQGFAR LSAIYGGTYM<br>251 LNKPIEEIIV QNGKVIGVKS EGEIARCKQL ICDPSYVKDR VEKVGQVIRV<br>301 ICILSHPIKN TNDANSCQII IPQNQVNRKS DIYVCMISFA HNVAAQGK**YI**<br>351 **AIVSTTVETK** EPEK**EIRPAL ELLEPIEQKF VSISDLLVPK DLGTESQIFI**<br>401 **SR**TYDATTHF ETTCDDIKNI YKRMTGSEFD FEEMKRKKND IYGED<br>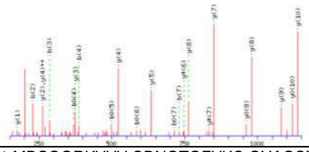
5	P61160	44,7	6,30	72	2	3%	ARP2	1 MDSQGRKVVV CDNGTGFVKC GYAGSNFPEH IFPALVGRPI IRSTTKVGNI<br>51 EIK**DLMVGDE ASELR**SMLEV NYPMENGIVR NWDDMKHLWD YTFGPEKLNI<br>101 DTRNCKILLT EPPMNPTKNR EKIVEVMFET YQFSGVYVAI QAVLTLYAQG<br>151 LLTGVVVDSG DGVTHICPVY EGFSLPHLTR RLDIAGRDIT RYLIKLLLLR<br>201 GYAFNHSADF ETVRMIKEKL CYVGYNIEQE QKLALETTVL VESYTLPDGR<br>251 IIKVGGERFE APEALFQPHL INVEGVGVAE LLFNTIQAAD IDTRSEFYKH<br>301 IVLSGGSTMY PGLPSRLERE LKQLYLERVL KGDVEKLSKF KIRIEDPPRR<br>351 KHMVFLGGAV LADIMKDKDN FWMTRQEYQE KGVRVLEKLG VTVR<br>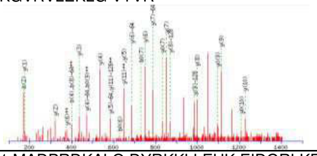
6	P62333	44,1	7,10	244	10	27%	PRS10	1 MADPRDKALQ DYRKKLLEHK EIDGRLKELR EQLKELTK**QY EKSENDLKAL**<br>51 **QSVGQIVGEV LK**QLTEEKFI VKATNGPRYV VGCRRQLDKS KLKPGTR**VAL**<br>101 **DMTTLTIMR**Y LPREVDPLVY NMSHEDPGNV SYSEIGGLSE QIR**ELREVIE**<br>151 **LPLTNPELFQ R**VGIIPPK**GC LLYGPPGTGK** TLLAR**AVASQ LDCNFLKVVS**<br>201 **SSIVDKYIGE SAR**LIR**EMFN YAR**DHQPCII FMDEIDAIGG RR**FSEGTSAD**<br>251 **R**EIQRTLMEL LNQMDGFDTL HRVKMIMATN RPDTLDPALL RPGRLDRKIH<br>301 IDLPNEQARL DILKIHAGPI TK**HGEIDYEA IVKLSDGFNG ADLRNVCTEA**<br>351 **GMFAIR**ADHD FVVQEDFMKA VRKVADSKKL ESKLDYKPV<br>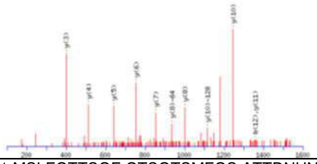
7	P78406	40,9	7,96	135	6	19%	RAE1L	1 MSLFGTTSGF GTSGTSMFGS ATTDNHNPMK DIEVTSSPDD SIGCLSFSPP<br>51 TLPGNFLIAG SWANDVRCWE VQDSGQTIPK AQQMHTGPVL DVCWSDDGSK<br>101 VFTASCDKTA KMWDLSSNQA IQIAQHDAPV KTIHWIKAPN YSCVMTGSWD<br>151 KTLKFWDTR**S SNPMMVLQLP ER**CYCADVIY PMAVVATAER **GLIVYQLENQ**<br>201 **PSEFR**RIESP LKHQHRCVAI FKDK**QNKPTG FALGSIEGRV AIHYINPPNP**<br>251 **AK**DNFTFKCH RSNGTNTSAP QDIYAVNGIA FHPVHGTLAT VGSDGR**FSFW**<br>301 **DKDAR**TKLKT SEQLDQPISA CCFNHNGNIF AYASSYDWSK GHEFYNPQKK<br>351 **NYIFLR**NAAE ELKPRNKK

								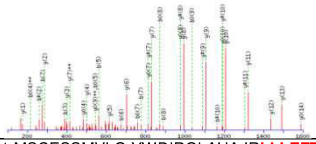
8	P09211	23,3	5,43	432	6	49%	GSTM3	1 MSCESSMVLG YWDIRGLAHA IR**LLLEFTDT SYEEKR**YTCG EAPDYDRSQW<br>51 LDVKFKLDLD FPNLPYLLDG KNK**ITQSNAI LR**YIARKHNM CGETEEEKIR<br>101 **VDIIENQVMD FR**TQLIRLCY SSDHEKLKPQ YLEELPGQLK QFSMFLGKFS<br>151 WFAGEKLTFV DFLTYDILDQ NRIFDPK**CLD EFPNLK**AFMC RFEALEKIAA<br>201 YLQSDQFCKM PINNKMAQWG NKPVC<br>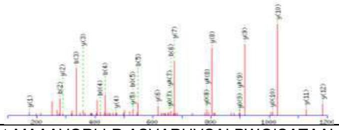
9	P30048	27,6	7,67	113	6	22%	PRDX3	1 MAAAVGRLLR ASVARHVSAI PWGISATAAL RPAACGRTSL TNLLCSGSSQ<br>51 AKLFSTSSSC HAPAVTQHAP YFKGTAVVNG EFK**DLSLDDF KGK**YLVLFFY<br>101 PLDFTFVCPT EIVAFSDKAN EFHDVNCEVV AVSVDSHFSH LAWINTPRKN<br>151 GGLGHMNIAL LSDLTKQISR **DYGVLLEGSG LALRGLFIID PNGVIKHLSV**<br>201 **NDLPVGRSVE ETLRLVK**AFQ YVETHGEVCP ANWTPDSPTI KPSPAASKEY<br>251 FQKVNQ<br>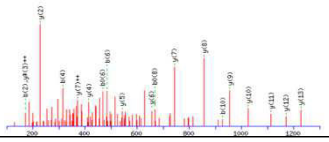
